# The developmental Wnt signaling pathway effector β-catenin/TCF mediates hepatic functions of the sex hormone estradiol in regulating lipid metabolism

**DOI:** 10.1371/journal.pbio.3000444

**Published:** 2019-10-07

**Authors:** Lili Tian, Weijuan Shao, Wilfred Ip, Zhuolun Song, Yasaman Badakhshi, Tianru Jin

**Affiliations:** 1 Division of Advanced Diagnostics, Toronto General Hospital Research Institute, University Health Network, Toronto, Canada; 2 Department of Physiology, University of Toronto, Toronto, Canada; 3 Banting and Best Diabetes Center, Faculty of Medicine, University of Toronto, Toronto, Canada; Hong Kong University, HONG KONG

## Abstract

The bipartite transcription factor β-catenin (β-cat)/T cell factor (TCF), formed by free β-cat and a given TCF family member, serves as the effector of the developmental Wnt signaling cascade. β-cat/TCFs also serve as effectors of certain peptide hormones or growth factors during adulthood. We reported that liver-specific expression of dominant-negative Transcription factor 7 like 2 (TCF7L2DN) led to impaired glucose disposal. Here we show that, in this *LTCFDN* transgenic mouse model, serum and hepatic lipid contents were elevated in male but not in female mice. In hepatocytes, TCF7L2DN adenovirus infection led to stimulated expression of genes that encode lipogenic transcription factors and lipogenic enzymes, while estradiol (E2) treatment attenuated the stimulation, associated with Wnt-target gene activation. Mechanistically, this E2-mediated activation can be attributed to elevated β-cat Ser675 phosphorylation and *TCF* expression. In wild-type female mice, ovariectomy (OVX) plus high-fat diet (HFD) challenge impaired glucose disposal and insulin tolerance, associated with increased hepatic lipogenic transcription factor sterol regulatory element-binding protein 1-c (*SREBP-1c*) expression. In wild-type mice with OVX, E2 reconstitution attenuated HFD-induced metabolic defects. Some of the attenuation effects, including insulin intolerance, elevated liver-weight gain, and hepatic *SREBP-1c* expression, were not affected by E2 reconstitution in HFD-fed *LTCFDN* mice with OVX. Finally, the effects of E2 in hepatocytes on β-cat/TCF activation can be attenuated by the G-protein-coupled estrogen receptor (GPER) antagonist G15. Our study thus expanded the scope of functions of the Wnt pathway effector β-cat/TCF, as it can also mediate hepatic functions of E2 during adulthood. This study also enriches our mechanistic understanding of gender differences in the risk and pathophysiology of metabolic diseases.

## Abstract 2

Certain hormones and growth factors are known to exert their effects via crosstalk with the Wnt signalling pathway. This study shows that in the liver, the Wnt pathway effector β-catenin/TCF (a bipartite transcription factor) mediates the role of the female hormone estrogen in maintaining lipid homeostasis, downstream of the G-protein coupled estrogen receptor.

## Introduction

Transcription factor 7 like 2 (TCF7L2, Tcf7l2 in rodents) and the other three T cell factor (TCF)/Tcf family members (Tcf7, Tcf7l1, and lymphoid enhancer binding factor 1 [Lef1]) are principal effectors of the canonical Wnt signalling cascade (referred to as the Wnt pathway hereafter) in mammals. When a TCF family member is partnered with the free β-catenin (β-cat) molecule, the formed bipartite transcription factor β-cat/TCF regulates expression of the Wnt pathway downstream target genes, in response to the stimulation by certain canonical Wnt ligands. Investigations from different disciplines have been continuing to uncover the complexity of this signaling cascade, including the existence of multiple Wnt ligands with common or unique functions, receptors, coreceptors, membrane-bound or soluble positive and negative regulators, alternative signaling pathways, and, even more importantly, the bidirectional feature of TCF family members [1–6].

Previous investigations by our team and by others have shown that certain peptide hormones and growth factors, such as insulin, insulin-like growth factor 1 (IGF-1), and glucagon-like peptide-1 (GLP-1), may exert their functions via cross-talking with the Wnt signaling cascade. This can be achieved via stimulating β-cat phosphorylation at the C-terminal Ser675 and Ser552 residues [1,7–11]. We have therefore suggested that the developmental Wnt signaling cascade effector β-cat/TCF is also among the effectors of certain peptide hormones and growth factors during adulthood [1,8].

We have reported that mouse or human hepatocytes express *TCF7*, *TCF7L1*, and *TCF7L2* but not *LEF1* [9]. Among them, *TCF7L2* has been recognized as a type 2 diabetes (T2D) risk gene, revealed by reproducible genome-wide association studies (GWASs) [1,12,13]. Despite intensive global effort for more than a decade, we are still unable to unite our view of whether *TCF7L2* variants in T2D risk subjects represent gain-of-function or loss-of-function events [1,14–16]. It still remains unknown whether these T2D risk TCF7L2 single nucleotide polymorphisms (SNPs) indeed affect TCF7L2 expression in any given metabolic organs [1]. A recent study suggested that the genomic location harbouring one of the SNPs, rs7903146, may serve as a regulatory region of the gene that encodes long-chain fatty acid CoA ligase 5 (ACSL5) [17]. Nevertheless, the investigations have deepened our knowledge of metabolic functions of Wnt signaling cascade in metabolic organs in general, with the generation and assessment of various transgenic mouse models [15,18–23].

To overcome the redundant function of TCFs and the bidirectional feature of a given TCF family member, we and others have adopted a powerful functional knockdown approach in conducting in vitro investigations as well as in generating transgenic mouse models [20,21,24–29]. In the *LTCFDN* mouse model that we have generated, expression of dominant-negative TCF7L2 (TCF7L2DN) is driven by the mouse albumin promoter [26]. The *LTCFDN* transgenic mice fed with a regular low-fat diet (LFD) exhibited progressive impairment on pyruvate and glucose tolerance, in the absence of the development of insulin resistance. Furthermore, *LTCFDN* hepatocytes showed elevated glucose production and attenuated response to in vitro Wnt-3a-treatment–induced repression on glucose production [26].

Wnt signaling cascade is also involved in lipid homeostasis. Certain mutations on the Wnt ligand coreceptor lipoprotein receptor-related protein 6 (LRP6) are associated with the susceptibility of dyslipidemia [30], whereas in mice, *Lrp6*-deficiency–induced hyperlipidemia can be partially reversed by Wnt-3a administration [31]. The direct involvement of β-cat/TCF on lipid homeostasis or hepatic lipogenesis, however, remains largely unknown.

In the current study, we found that male *LTCFDN* mice exhibited elevated triglyceride (TG) levels and increased hepatic lipid deposition. Such increases were absent in female *LTCFDN* mice, suggesting that female hormones might be able to overcome functional attenuation on β-cat/TCF activity. Utilizing the ovariectomy (OVX) surgery procedure along with high-fat diet (HFD) feeding and the female hormone estradiol (E2) reconstitution, we obtained evidence that the attenuation effect of E2 on insulin intolerance and hepatic lipogenic program activation in response to HFD challenge requires the intact Wnt/β-cat signaling cascade in the liver.

## Results

### Male *LTCFDN* mice show elevated hepatic and serum TG levels and liver tissue fat deposition

**Fig 1:**
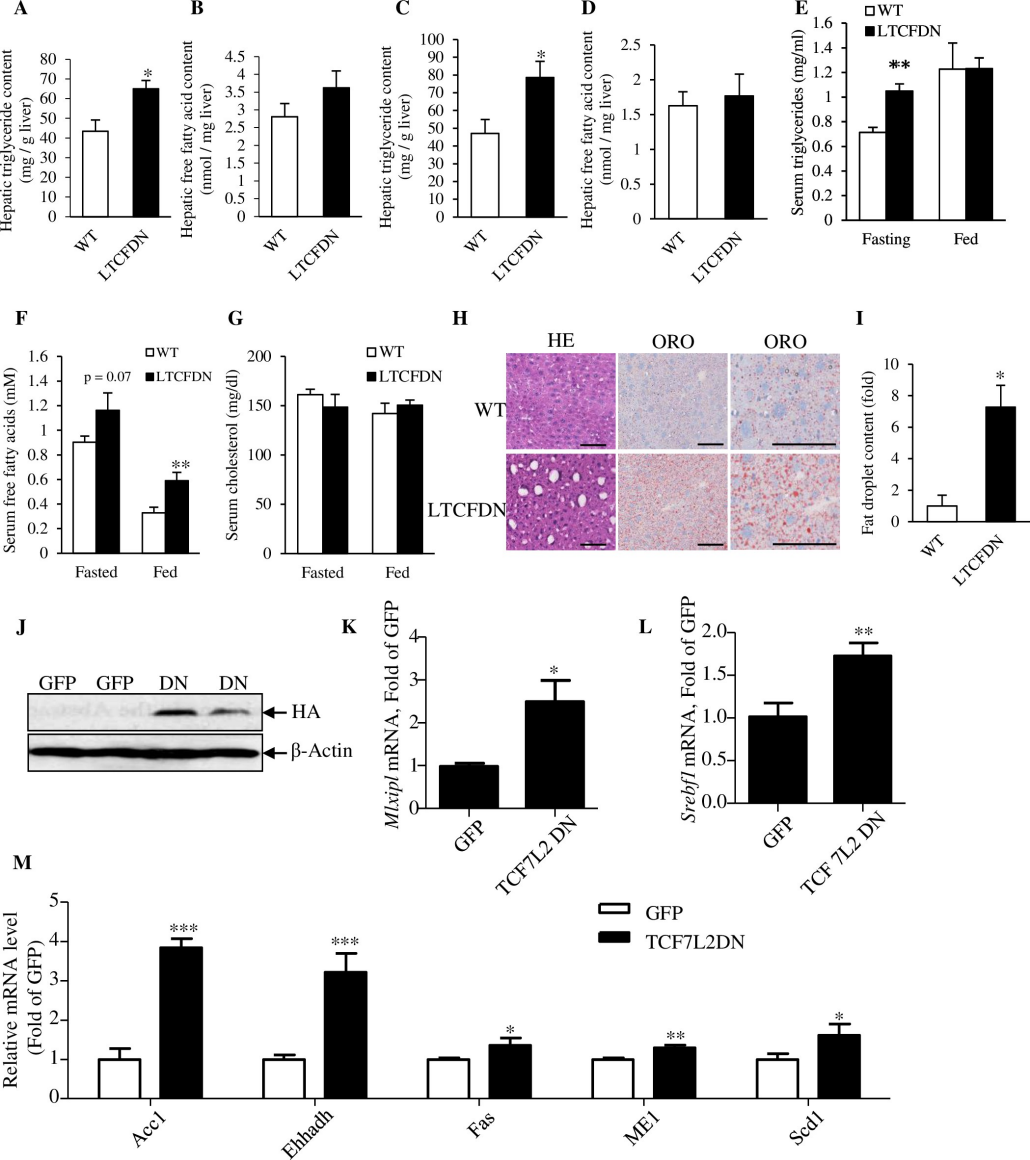
Male *LTCFDN* mice show increased hepatic lipid content while Ad-TCF7L2DN infection increases hepatic lipogenic gene expression. (A–B) Hepatic TG and FFA contents in 2-week-old mice (*n* = 6 for WT and *LTCFDN* mice; gender of the mice was not determined). (C–D) Hepatic TG and FFA contents in 12-week-old male mice. (E–G) Serum TG, FFA, and cholesterol levels in 12-week-old male mice. (H) HE and ORO staining in adult (24 weeks) mouse liver sections. Scale bar, 100 μm. (I) Quantification of ORO staining in panel H. (J) HA-tagged TCF7L2DN detection in MPHs 48 hours after Ad-TCF7L2DN infection. (K–L) *ChREBP* (*Mlxipl*) and *SREBP*-1c (*Srebf1*) mRNA detection in Ad-TCF7L2DN infected MPHs. (M) ChREBP and SREBP-1c downstream genes mRNA levels in Ad-TCF7L2DN-infected MPH. The 5 genes include those that encode Acc1, Ehhadh, Fas, Me1, and Scd1. *p < 0.05; **p < 0.01; ***p < 0.001. Values represent mean ± SEM. For panels C–G, *N* ≥ 4. Panel H are representative images. For Panel I and K–M, *N* ≥ 3. Underlying numerical values can be found in S1 Data. Acc1, acetyl-coA carboxylase; Ad-TCF7L2DN, adenovirus that expresses TCF7L2DN; ChREBP, carbohydrate-responsive element-binding protein; Ehhadh, peroxisomal L-bifunctional enzyme; Fas, fatty acid synthase; FFA, free fatty acid; GFP, green fluorescence protein; HA, hemagglutinin tag; HE, hematoxylin–eosin; Me1, cytosolic malic enzyme 1; MPH, mouse primary hepatocyte; ORO, oil red O; Scd1, stearoyl-coA desaturase-1; SREBP-1c, sterol regulatory element-binding protein 1-c; TCF7L2DN, dominant-negative TCF7L2; TG, triglyceride; WT, wild-type.

To determine whether the Wnt signaling pathway effector β-cat/TCF is also involved in hepatic lipid homeostasis, we have first of all examined several lipid parameters in *LTCFDN* mice fed with the regular LFD. In a set of 2-week-old *LTCFDN* mice (regardless of their genders), we observed that hepatic TG content was increased (Fig 1A) while their hepatic free fatty acid (FFA) level showed a trend of increase (Fig 1B). The 2-week-old mice, however, carried no appreciable “abnormalities” on their body weight, liver weight, random glucose, serum TG, FFA, or cholesterol levels (S1 Fig).

In 12-week-old male *LTCFDN* mice, we found that hepatic TG content was elevated when compared with that in age- and sex-matched littermate controls (Fig 1C), although their hepatic FFA levels were comparable (Fig 1D). Male *LTCFDN* (12 weeks old) also showed elevated serum TG levels during fasting but not after feeding (Fig 1E), in association with lack of a defect on intraperitoneal insulin tolerance test (IPITT) that we have reported previously [26]. Serum FFA level was increased significantly after feeding and with a trend of elevation after fasting (Fig 1F). Serum cholesterol levels were comparable in male *LTCFDN* and sex-matched wild-type mice (Fig 1G). Lipid deposition in the liver tissue sections of *LTCFDN* male mice (24 weeks) was greatly increased compared with that in sex- and age-matched littermate controls (Fig 1H and 1I). Thus, liver-specific functional knockdown of the Wnt signaling cascade with a dominant-negative TCF molecule resulted in elevated serum and hepatic lipid contents and increased hepatic lipid deposition. We also found that hepatic glycogen content in 12-week-old male *LTCFND* mice showed a trend of increase, whereas in mouse primary hepatocytes (MPHs), adenovirus Ad-TCF7L2DN infection but not Ad-TCF7L2 infection increased expression of glycogen synthase 2 (Gys2) (S2 Fig).

### TCF7L2DN expression increases lipogenic gene expression

Taking advantage of possessing the TCF7L2DN adenovirus [26], we then conducted further functional knockdown experiments in vitro in the human HepG2 cell line and in MPH. Cells were infected with either the control virus green-fluorescence-protein-expressing adenovirus (Ad-GFP) or Ad-TCF7L2DN, verified by western blotting for hemagglutinin (HA) tag detection (Fig 1J and S3A Fig). Forty-eight hours after virus infection, cells were harvested for assessing lipogenic gene expression. As shown (S3B and S3C Fig), in HepG2 cells following Ad-TCF7L2DN infection, expression of genes that encode the 2 key lipogenic transcription factors—carbohydrate-responsive element-binding protein (*ChREBP*, *Mlxipl*) and sterol regulatory element-binding protein 1-c (*SREBP-1c*, *srebf1*)—were increased. In MPH, Ad-TCF7L2DN infection also increased expression of both *Mlxipl* and *Srebf1* (Fig 1K and 1L), accompanied by increased expression of hepatic genes that encode the 5 lipogenic enzymes (Fig 1M), known as acetyl-coA carboxylase (*Acc1*), enoyl-coA hydratase and 3-hydroxyacyl coA dehydrogenase (also known as peroxisomal L-bifunctional enzyme [*Ehhadh*]), fatty acid synthase (*Fas*), cytosolic malic enzyme 1 (*Me1*), and stearoyl-coA desaturase-1 (*Scd1*).

### Female *LTCFDN* mice show virtually no elevation on serum and hepatic lipid contents

**Fig 2:**
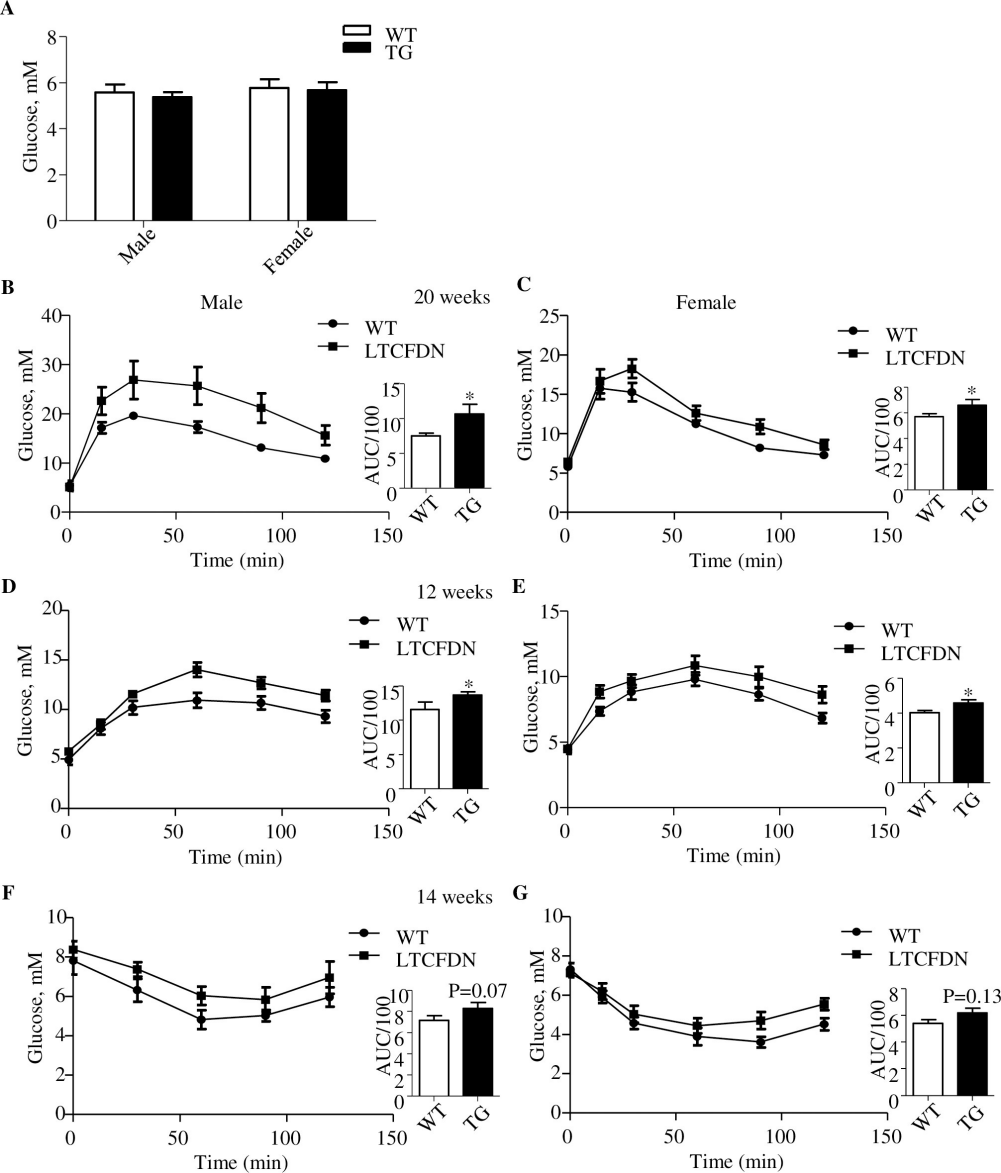
Female and male *LTCFDN* mice show comparable impairment on glucose and pyruvate tolerance. (A) Fasting glucose levels. *N* = 6 for each of the subgroups. (B–C) IPGTT in 20-week-old *LTCFDN* and WT mice. (D–E) IPPTT in 12-week-old *LTCFDN* and WT mice. (F–G) IPITT in 14-week-old *LTCFDN* and WT mice *p < 0.05; **p < 0.01. Values represent mean ± SEM. *N* = 3–7 for panels B–G. Underlying numerical values can be found in S1 Data. AUC, area under the curve; IPGTT, intraperitoneal glucose tolerance test; IPITT, intraperitoneal insulin tolerance test; IPPTT, intraperitoneal pyruvate tolerance test; SEM, standard error of the mean; WT, wild-type.

As the crosstalk between the Wnt/β-cat/TCF signaling cascade and the estrogen signaling has been indicated in both cancer and developmental biology fields [32, 33], we determined to assess whether the defect on glucose disposal and lipid metabolism observed in male *LTCFDN* mice is different in female *LTCFDN* mice. As shown in Fig 2A, without HFD challenge, male and female *LTCFDN* mice had comparable fasting blood glucose levels. We then conducted the 3 common intraperitoneal metabolic tolerance tests. As shown (Fig 2B and 2C), both male and female *LTCFDN* mice (20 weeks) exhibited impaired glucose disposal after glucose challenge, although it appeared that the impairment was slightly worse in male mice. Impaired pyruvate tolerance was also observed in both male and female *LTCFDN* mice (Fig 2D and 2E). In agreement with our previous observations [26], *LTCFDN* mice on an LFD show a lack of insulin intolerance, assessed by IPITT, regardless of gender (Fig 2F and 2G).

**Fig 3:**
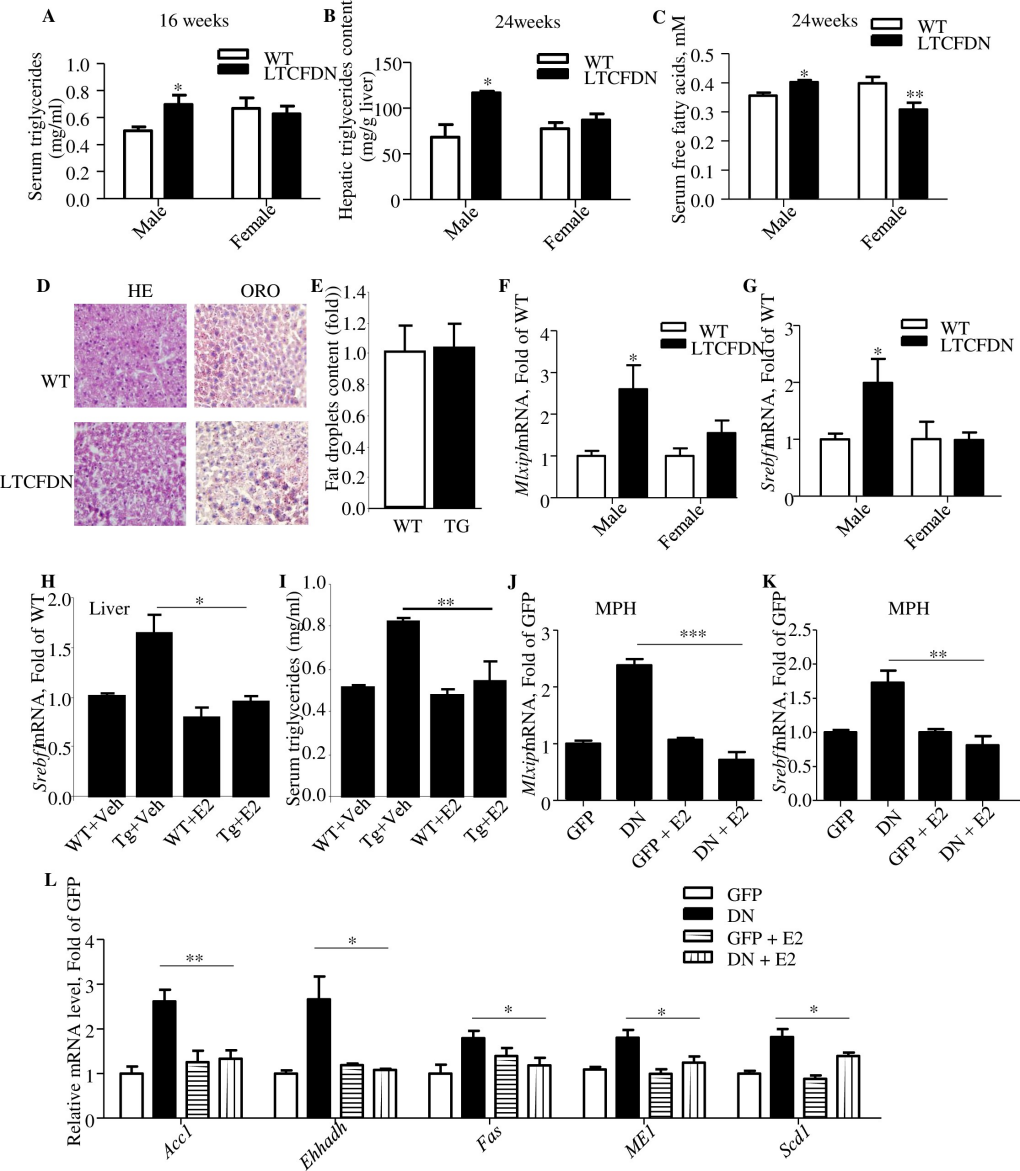
Female *LTCFDN* mice show virtually no defect on serum and hepatic lipid contents while Ad-TCF7L2DN infection-induced lipogenic gene expression can be attenuated by E2. (A–C) Serum and hepatic TG contents and serum FFA levels. (D–E) HE and ORO staining of liver section from 20-week-old female *LTCFDN* mice. Panel E shows the results of quantitative analysis of panel D. *N* = 5. (F–G) Hepatic *ChREBP* (*Mlxipl*) and *SREBP-1c* (*Srebf1*) mRNA levels in 24-week-old mice. (H–I) Hepatic *SREBP-1c* mRNA and fasting plasma TG levels in male *LTCFDN* received daily E2 injection for 6 days. (J–L) *ChREBP* (*Mlxipl*), *SREBP-1c* (*Srebf1*) and their downstream gene mRNA levels in E2-treated and Ad-TCF7L2DN (DN)-infected MPH. *p < 0.05; **p < 0.01; ***p < 0.0001. *N* ≥ 3 for all panels. Values represent mean ± SEM except for panel H and I, in which values represent meant ± SD. Underlying numerical values can be found in S1 Data. ACC, acetyl-coA carboxylase; Ad-TCF7L2DN, adenovirus that expresses TCF7L2DN; ChREBP, carbohydrate-responsive element-binding protein; DN, dominant negative; E2, estradiol; FFA, free fatty acid; EHHADH, enoyl-coA hydratase and 3-hydroxyacyl coA dehydrogenase; Fas, fatty acid synthase; GFP, green fluorescence protein; HE, hematoxylin–eosin; ME1, cytosolic malic enzyme 1; MPH, mouse primary hepatocyte; ORO, oil red O; SCD-1, stearoyl-coA desaturase-1; SEM, standard error of the mean; SREBP-1, sterol regulatory element-binding protein 1-c; TG, triglyceride; Veh, vehicle; WT, wild type.

We then compared hepatic and serum TG levels in male and female *LTCFDN* mouse littermates. As shown, male *LTCFDN* mice showed elevated serum (Fig 3A) and hepatic (Fig 3B) TG contents. However, comparable plasma and hepatic TG levels were found in age-matched female littermate mice, regardless of their genotypes (Fig 3A and 3B). Fig 3C shows that random serum FFA level in female *LTCFDN* mice was also not higher than that in sex-matched wild-type littermate controls. Furthermore, increased liver lipid deposition was also not observed in female *LTCFDN* mice (24 weeks, Fig 3D and 3E). Finally, increased hepatic *ChREBP* (*Mlxipl*) and *SREBP-1c* (*Srebf1*) expression observed in male *LTCFDN* mouse liver was also not observed in female *LTCFDN* mouse liver (Fig 3F and 3G). These observations collectively suggest that female mice are more tolerant to hepatic TCF7L2DN-expression–induced Wnt signaling cascade functional knockdown on lipid homeostasis.

To assess the involvement of the female hormone E2, we then conducted daily E2 injection in 6-week-old male *LTCFDN* mice for 6 days, with age- and sex-matched wild-type littermates as controls. As shown, the injection attenuated hepatic *SREBP-1c* mRNA level, associated with attenuated fasting serum TG level (Fig 3H and 3I).

### TCF7L2DN-induced lipogenic gene expression in hepatocytes can be attenuated by E2 treatment

The human HepG2 cell line was then infected with either the control virus Ad-GFP or Ad-TCF7L2DN. Forty-eight hours after virus infection, cells were treated with or without 100 nM E2 for 6 hours, followed by cell harvesting and quantitative reverse transcription polymerase chain reaction (qRT-PCR) analyses. As shown (S4 Fig), TCF7L2DN-induced *Mlxipl* and *Srebf1* elevations can be attenuated by E2 treatment. In Ad-GFP–infected cells, E2 treatment generated no appreciable effect on expression of these 2 lipogenic transcription factors.

The same adenovirus infection experiment was then conducted in MPH. Fig 3J and 3K show the similar attenuation effect of E2 treatment on TCF7L2DN-induced *Mlxipl* and *Srebf1* gene expression in MPH. With Ad-GFP infection, E2 treatment generated no appreciable effect on their expression as well. Consistently, TCF7L2DN-expression–induced expression of the 5 lipogenic enzyme genes were also attenuated by E2 treatment in MPH (Fig 3L). Thus, in response to β-cat/TCF functional knockdown, the female hormone E2 can exert a rescue effect in the liver on the abnormal activation of the lipogenic program. Furthermore, we observed that, in MPH, *Mlxipl* expression induced by high glucose (20 mM) can also be blocked by E2 treatment and the lack of synergistic effect of high glucose and TCF7L2DN expression on stimulating *Mlxipl* expression (S5 Fig).

### E2 treatment increases β-cat Ser675 phosphorylation and *Tcf* gene expression

**Fig 4:**
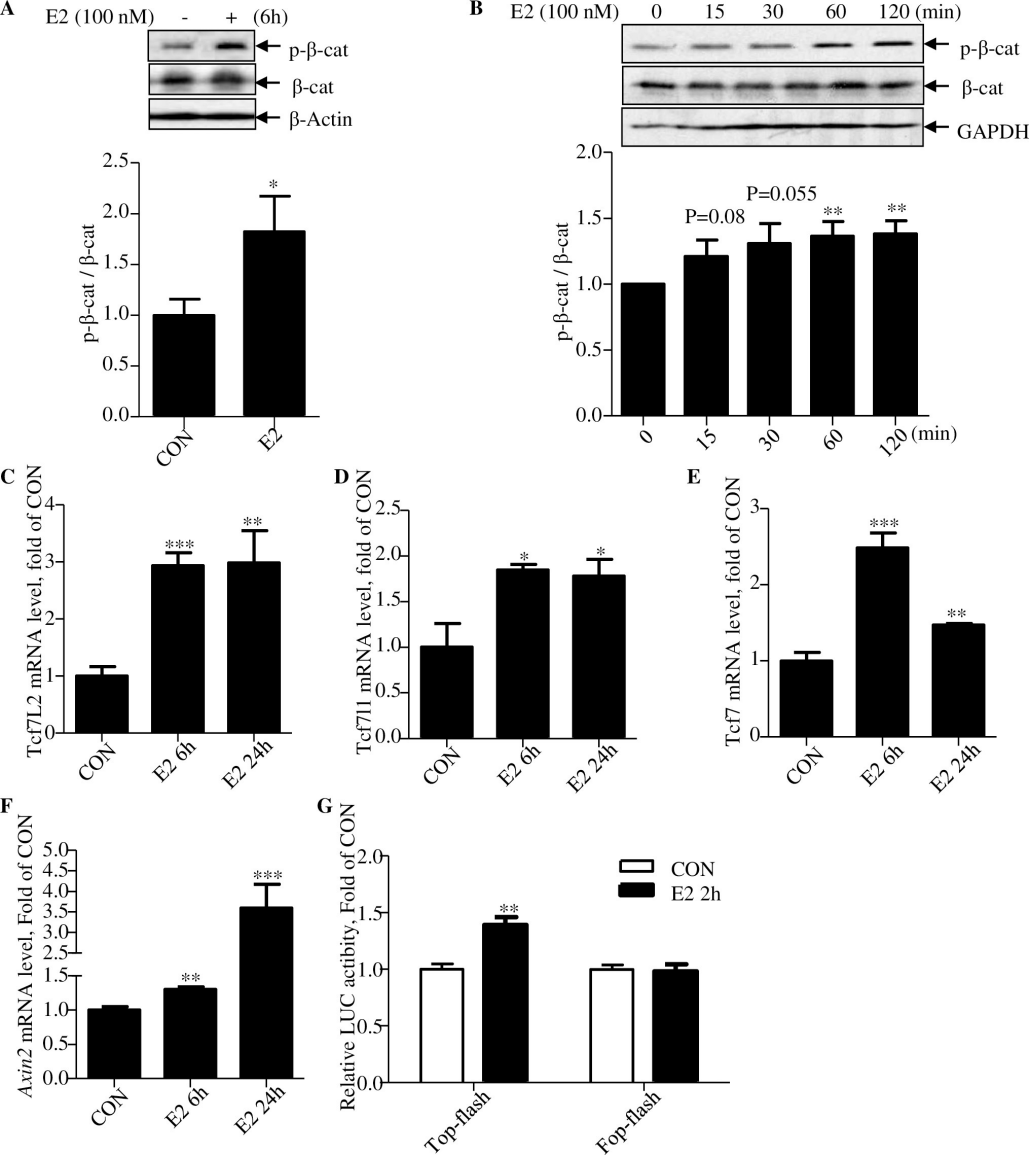
E2 treatment increases β-cat S675 phosphorylation and expression of the 3 Tcf family members. (A–B) The phosphorylation (Ser675) level of β-cat after indicated time and dose of E2 treatment in MPH. (C) *Tcf7l2*, (D) *Tcf7l1*, (E) *Tcf7*, and (F) *Axin2* mRNA levels after E2 treatment for 6 hours and 24 hours in MPH. (G) The expression of the Top/Fop-flash LUC reporter after E2 treatment. *p < 0.05; **p < 0.01, ***p < 0.0001. *N* ≥ 3 for all panels. Values represent mean ± SEM. Underlying numerical values can be found in S1 Data. β-cat, β-catenin; Axin2, axis inhibition protein 2; CON, control; E2, estradiol; LUC, luciferase; MPH, mouse primary hepatocyte; SEM, standard error of the mean; Tcf, T cell factor.

To explore the underlying mechanism for the rescue effect of E2 treatment in response to TCF7L2DN-mediated functional knockdown; we tested the in vitro effect of E2 in MPH. We found that 6-hour E2 treatment stimulated β-cat Ser675 phosphorylation (Fig 4A), and the stimulation increased along with the treatment time within the 120-minute experimental time (Fig 4B). E2 treatment also transiently increased cAMP response element-binding protein (CREB) phosphorylation (Ser133) (at 15 minutes, S6 Fig). Fig 4C–4E shows that in MPH, 6-hour E2 treatment resulted in elevated mRNA levels of the 3 Tcf gene members, *Tcf7l2*, *Tcf7l1*, and *Tcf7*, which are known to be expressed in the mouse liver [9], and the elevation sustained for 24-hour experimental period. Importantly, the elevations were associated with increased expression of axis inhibition protein 2 (*Axin2*), a known Wnt signaling pathway downstream target gene, especially after 24-hour E2 treatment (Fig 4F). Finally, we found that E2 treatment increased the activity of the β-cat/TCF responsive Top-Flash luciferase (LUC) fusion gene plasmid, although the activation appeared relatively modest in the utilized 293 naïve cell system (Fig 4G).

### OVX accelerates HFD-induced metabolic defects

**Fig 5:**
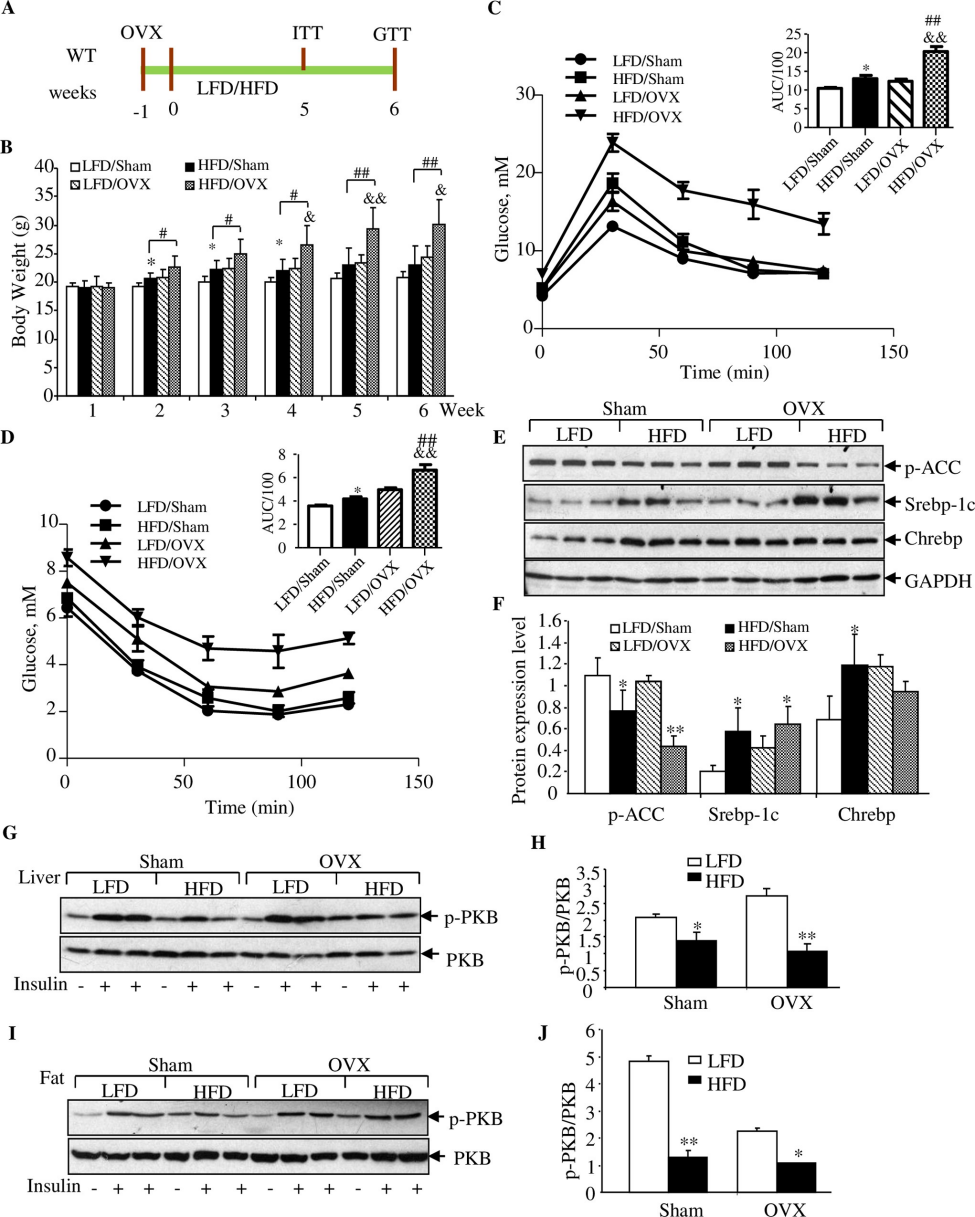
OVX accelerates HFD-induced body weight gain, defect on glucose disposal, and hepatic lipogenic gene expression. (A) Illustration of the experimental timeline. OVX or sham surgery was performed on mice at the age of 5 weeks. After 1-week recovery, mice were fed with LFD or HFD for 6 weeks, with IPITT and IPGTT performed on indicated time. (B) Weekly body weight recording. (C–D) IPITT and IPGTT performed at fifth week and sixth week, respectively. “*” indicates LFD/Sham versus HFD/Sham; “&” indicates LFD/OVX versus HFD/OVX; “§” indicates LFD/Sham versus LFD/OVX; “#” indicates HFD/Sham versus HFD/OVX. (E) Western blotting shows the detection of p-ACC (Ser79), SREBP-1c, and ChREBP in the indicated group of mice. GAPDH is the house keep protein. (F) Densitometrical analysis of panel E. (G–J) Western blotting show the detection of PKB S473 phosphorylation in the liver (G–H) and fat (I–J) in response to insulin i.p. injection in indicated group of mice. Mice were injected with PBS or insulin 10 minutes before their euthanization. *N* = 3–5 for panels B–D. Values represent mean ± SD. For panels E–J, *N* = 3. Underlying numerical values can be found in S1 Data. AUC, area under the curve; ChREBP, carbohydrate-responsive element-binding protein; GAPDH, glyceraldehyde-3-phosphate dehydrogenase; HFD, high-fat diet; i.p., intraperitoneal; IPGTT, intraperitoneal glucose tolerance test; IPITT, intraperitoneal insulin tolerance test; LFD, low-fat diet; OVX, ovariectomy; p-ACC, phosphorylated acetyl-coA carboxylase; PKB, protein kinase B; SREBP-1c, sterol regulatory element-binding protein 1-c.

Intensive previous investigations in rodent species have attributed the effect of E2 on attenuating HFD-induced body weight gain mainly to the function of this female hormone in adipose tissues, the muscle tissue, and the brain [34–36]. To investigate the involvement of the liver, we have first of all conducted a set of experiments in wild-type female mice with HFD feeding and the OVX surgery (Fig 5A). Fig 5B shows that with the sham surgery, HFD feeding increased body weight from the second to the fourth week, but not after the fifth week. In LFD-fed mice, OVX surgery itself (without HFD challenge) increased the body weight starting from the second week. Importantly, OVX surgery significantly increased the body weight of mice that received HFD challenge, starting at the second week and continuing during the entire 6-week experimental period. These observations collectively suggest that, unlike male mice, female mice possess a mechanism in protecting body weight gain in response to HFD consumption, and the mechanism involves the ovarian gland.

IPITT and intraperitoneal glucose tolerance test (IPGTT) were then conducted during the fifth week and the sixth week, respectively. As shown, impaired glucose disposal and insulin tolerance were observed for HFD-fed mice with the OVX surgery (Fig 5C and 5D). OVX surgery itself or HFD feeding also slightly impaired glucose and insulin tolerance (Fig 5C and 5D). Fig 5E and 5F show the effect of HFD challenge and OVX surgery on attenuating ACC Ser79 phosphorylation as well as on affecting SREBP-1c and ChREBP protein levels, while Fig 5G–5J as well as S7A and S7B Fig show the attenuation effect of HFD and OVX on insulin-stimulated PKB/Akt Ser473 phosphorylation in the liver, abdominal fat tissue, and in the skeletal muscles.

### The restoration effects of E2 reconstitution are impaired in *LTCFDN* mice

**Fig 6:**
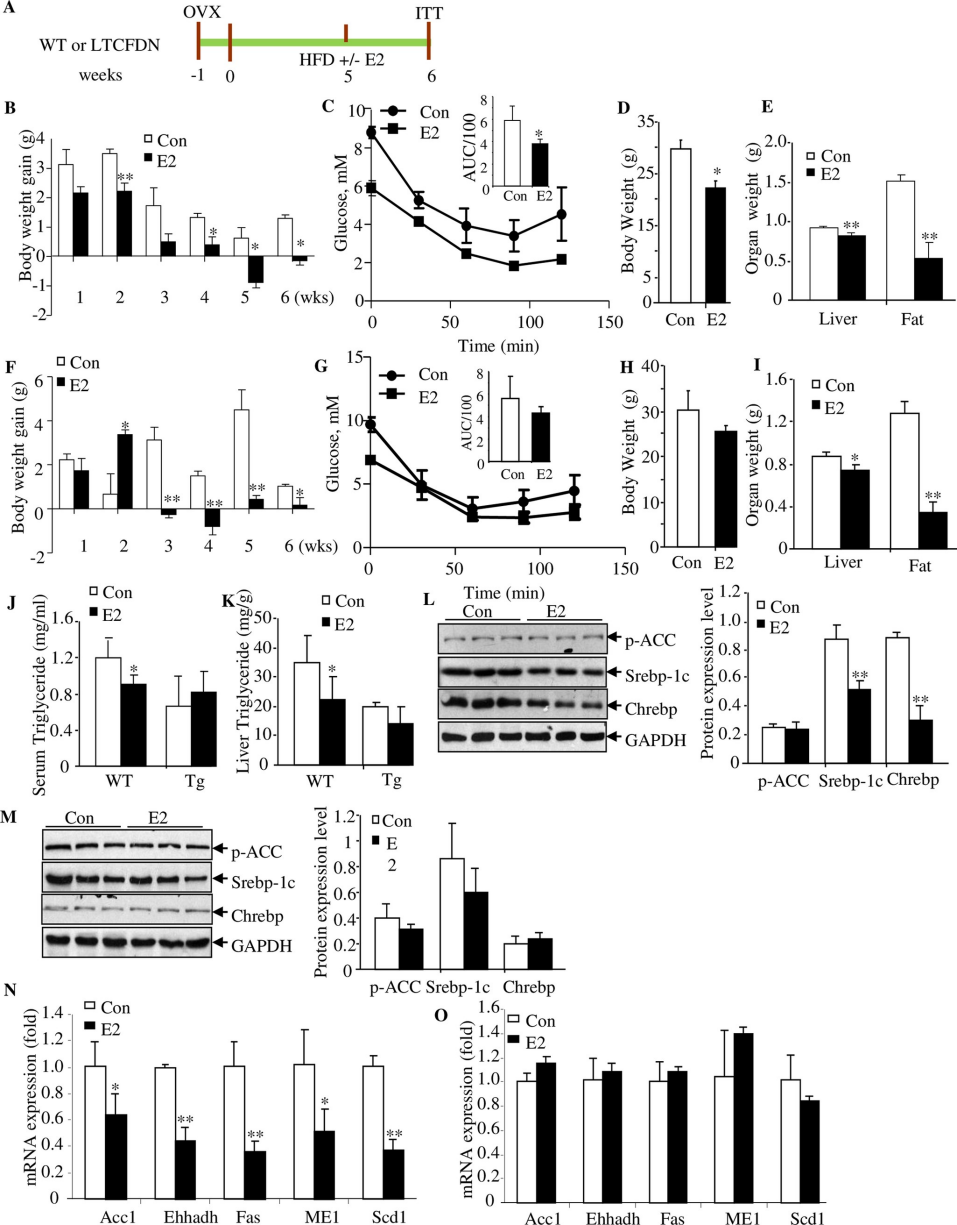
The restoration effect of E2 administration in HFD-challenged mice with OVX are impaired in *LTCFDN* mice. (A) Illustration of the experimental timeline. OVX was performed on WT or *LTCFDN* mice at the age of 5 weeks. After 1-week recovery, mice were fed with HFD, with or without E2 injection. (B–E) Comparison of weekly recorded body weight gain (panel B), IPITT (panel C), final-day body weight (panel D), and the liver as well as fat weight (panel E) in OVX-treated and HFD-challenged WT littermate controls, with or without E2 injection. (F–I) The above assessments in *LTCFDN* female mice. (J–K) Serum and hepatic TG content in indicated group of mice. (L–M) Western blotting shows the detection of p-ACC (Ser79), SREBP-1c and ChREBP in OVX treated and HFD-challenged WT mice (L) or *LTCFDN* (M), with and without E2 reconstitution. (N–O) Comparison of the 5 lipogenic gene mRNA expression in OVX treated and HFD-challenged WT (N) or *LTCFDN* (O) mice without and with E2 administration. *N* = 3–5 for panels B–O. Values represent mean ± SD. Underlying numerical values can be found in S1 Data. ACC, acetyl-coA carboxylase; ChREBP, carbohydrate-responsive element-binding protein; CON, control; E2, estradiol; EHHADH, enoyl-coA hydratase and 3-hydroxyacyl coA; Fas, fatty acid synthase; GAPDH, glyceraldehyde-3-phosphate dehydrogenase; HFD, high-fat diet; IPITT, intraperitoneal insulin tolerance test; ME1, cytosolic malic enzyme 1; OVX, ovariectomy; p-ACC, phosphorylated acetyl-coA carboxylase; SCD-1, stearoyl-coA desaturase-1; SREBP-1c, sterol regulatory element-binding protein 1-c; TG, triglyceride; WT, wild-type.

In order to determine the pathophysiological importance of the crosstalk between E2 and β-cat/TCF in the liver, we have then directly compared the restoration effect of E2 administration in wild-type and *LTCFDN* female mice after HFD challenge and OVX surgery, with assays illustrated in Fig 6A. In wild-type littermates, E2 administration attenuated the effect of OVX surgery and HFD challenge on body weight gain, started at the second week (Fig 6B), improved glucose disposal (assessed by IPITT at the sixth week) (Fig 6C), reduced the final-day body weight, and reduced the liver as well as the abdominal fat weight (Fig 6D and 6E). In *LTCFDN* mice, the attenuation effect of E2 on body weight gain was found to be delayed until the third week (Fig 6F). More importantly, E2 administration did not effectively restore the defect on glucose disposal, assessed by IPITT (Fig 6G), and had no appreciable effect on reducing the final-day body weight, although E2 administration also reduced the liver weight as well as abdominal fat volume in *LTCFDN* mice (Fig 6H and 6I). Fig 6J and 6K show that E2 administration did not attenuate serum or hepatic TG levels in *LTCFDN* as it did in the wild-type littermates, although absolute hepatic TG levels were not elevated in the transgenic mice at the 6-week experimental ending stage. Serum and hepatic FFA levels, however, were comparable in wild-type littermates and *LTCFDN* mice, regardless of systematic E2 administration (S8 Fig). In wild-type littermate controls, E2 administration attenuated hepatic SREBP-1c and ChREBP expression levels (Fig 6L), while such attenuation was not observed in *LTCFDN* female mice with OVX surgery (Fig 6M). Furthermore, we assessed expression of the 5 hepatic lipogenic enzyme genes in these mice. As shown, their expression levels were reduced in wild-type littermates but not in *LTCFDN* mice, following HFD challenge, OVX, and E2 administration (Fig 6N and 6O).

### The metabolic effects of E2 in cultured hepatocytes can be blocked by the G-protein-coupled estrogen receptor antagonist

**Fig 7:**
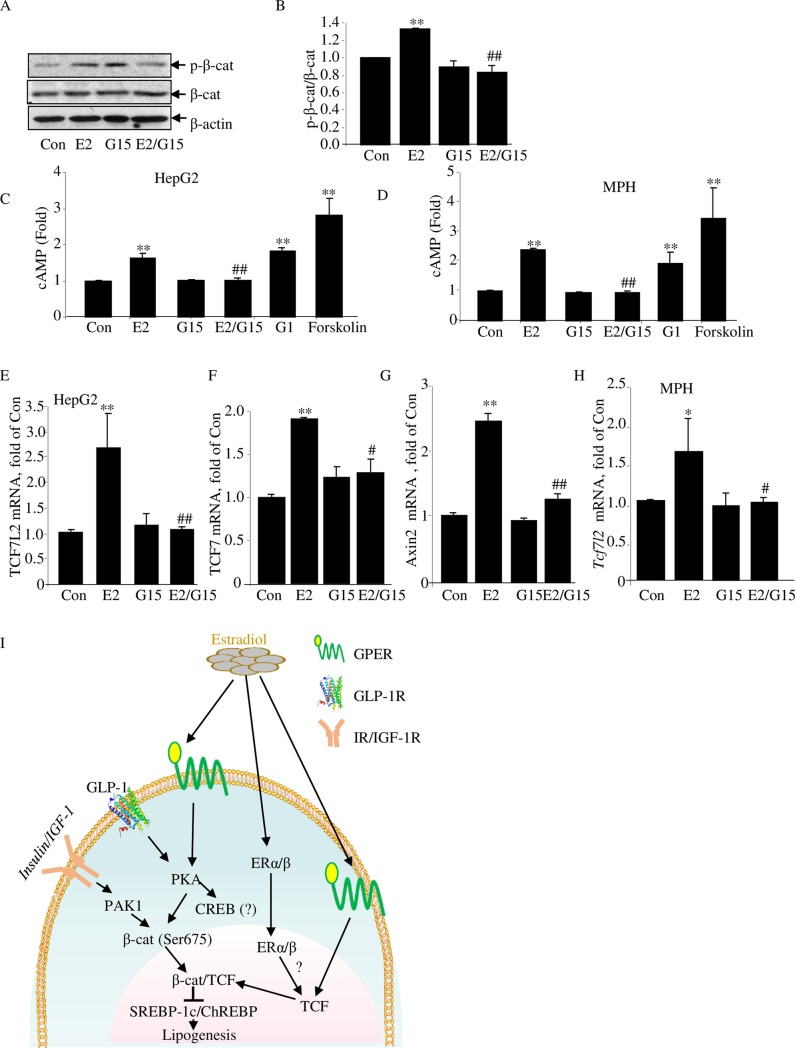
The GPER antagonist G15 blocks the effect of E2 in hepatocytes. (A–B) Western blot shows the effect of E2 and G15 treatment on β-cat Ser675 phosphorylation. Representative blot of 3 independent experiments, with densitometrical analysis results presented in panel B. Cells were pretreated with or without G15 (10 nM) for 45 minutes, followed by E2 (100 nM) or vehicle treatment (as control) for another 120 minutes. (C–D) Cellular cAMP levels, determined by ELISA, in the HepG2 cell line (C) and in MPH (D). Cells were pretreated with IBMX (10 μM) or IMBX plus G15 (10 nM) for 15 minutes, followed by further E2 (100 nM) or G1 (100 nM), or forskolin (10 μM) treatment for 15 minutes. Data are presented as fold change against that of the control samples. (E–G) qRT-PCR shows *TCF7L2*, *TCF7*, and *Axin2* mRNA levels in HepG2 cells with designated treatment. Cells were pretreated with or without G15 (10 nM) for 45 minutes, followed by further E2 (100 nM) or vehicle treatment for 6 hours. (H) qRT-PCR shows *TCF7L2* levels in MPHs with designated treatment. Cells were pretreated with or without G15 (10 nM) for 45 minutes, followed by further E2 (100 nM) or vehicle treatment for 6 hours. *N* ≥ 3 for panels A–H. Values represent mean ± SD. (I) A diagram summarizes the current understanding on the role of E2 on lipid metabolism. Underlying numerical values can be found in S1 Data. β-cat, β-catenin; Axin2, axis inhibition protein 2; CON, control; E2, estradiol; ERα/β, estrogen receptor α/β; ELISA, enzyme-linked immunosorbent assay; GLP-1, glucagon-like peptide-1; GLP-1R, glucagon-like peptide-1 receptor; GPER, G-protein-coupled estrogen receptor; IBMX, 3-isobutyl-1-methylxanthine; IGF-1R, Insulin-like growth factor 1 receptor; IR, insulin receptor; MPH, mouse primary hepatocyte; PAK1, p21-activated protein kinase 1; PKA, protein kinase A; qRT-PCR, quantitative reverse transcription polymerase chain reaction; TCF, T cell factor.

In addition to the nuclear receptors ERα and ERβ, estrogens, including E2, may utilize G-protein-coupled estrogen receptor (GPER; also known as G-protein-coupled receptor 30 [GPR30]) to exert its regulatory effects [37]. The nongenomic effects of E2, mediated by GPER, include adenylyl cyclase (AC) activation and cAMP elevation in the liver [38]. We have therefore tested the possible involvement of GPER with a GPER antagonist and an agonist. As shown in HepG2 cells, E2 treatment (100 nM for 120 minutes)–stimulated β-cat Ser675 phosphorylation can be blocked by the GPER antagonist G15 (Fig 7A and 7B). In conducting this experiment, we also observed a trend increase of CREB Ser133 phosphorylation level by 120-minute E2 treatment, while G15 treatment attenuated the increase (S9 Fig). In HepG2 cells as well as in MPH, E2 treatment increased cellular cAMP levels, while the increase was blocked by G15 treatment (Fig 7C and 7D). Furthermore, the GPER agonist G1 treatment showed a similar stimulatory effect on cellular cAMP elevation to that of E2 treatment (Fig 7C and 7D).

In response to a challenge, E2 may control energy intake and improve insulin signaling via its effects in the brain, adipose tissue, skeletal muscles, pancreatic islets, macrophages, and elsewhere, as documented in the literature. In hepatocytes, functional attenuation of β-cat/TCF activity leads to lipogenic program activation. E2, however, can activate β-cat, possibly via GPER, followed by protein kinase A (PKA) activation and increased β-cat Ser675 phosphorylation. E2 can also stimulate expression of the 3 TCF family members, via yet to be determined mechanisms with the participation of estrogen receptor α/β (ERα/β) or GPER, or both. The combined effect on β-cat post-translational modification and TCF gene expression leads to the repression of lipogenic transcription factor expression and reduced hepatic lipogenesis.

We then tested whether E2-stimulated TCF expression can be attenuated by G15 pretreatment. In the HepG2 cell line, G15 itself had no appreciable effect on *TCF7L2* or *TCF7* mRNA expression, while it blocked the stimulatory effect of E2 treatment (Fig 7E and 7F). Similarly, E2-stimulated *Axin2* expression was also blocked by G15 pretreatment (Fig 7G). The blockage effect of G15 on E2-stimulated *Tcf7l2* was then observed in MPH (Fig 7H). These observations collectively suggest the involvement of GPER in mediating the effect of E2 on β-cat/TCF activity, although we cannot exclude the potential involvement of ERα/β at this stage.

## Discussion

Although the Wnt signaling pathway was initially discovered in embryonic development and cancer biology studies [39–42], components of this signaling cascade are present in different cell lineages in adulthood. Evidently, this signaling cascade also participates in other important physiological and pathophysiological events during adulthood. β-cat/TCF, formed by β-cat and a given TCF member, is evidently involved in pancreatic islet β-cell genesis and proliferation, glucose-stimulated insulin secretion, and cholesterol metabolism, as well as the production and function of the incretin hormones GLP-1 and gastric inhibitory polypeptide (GIP) [1,7,43–45]. Furthermore, Wnt signaling cascade was shown to positively regulate osteogenesis but negatively regulate adipogenesis [24,46]. However, it remains difficult to clearly assign a physiological function to a given TCF family member because a given cell lineage always expresses multiple TCF members [1].

Following the discovery that *TCF7L2* is a T2D risk gene by GWAS [12,13], great efforts have been made to define its metabolic functions in various metabolic organs, including pancreatic islets, liver, brain, and adipose tissues. A few studies have suggested that T2D risk TCF7L2 SNPs represent gain-of-function events and TCF7L2 may stimulate hepatic gluconeogenesis [14,15,19]. Other investigations, including a few of them conducted by our team, however, presented a completely opposite view. Utilizing both cell culture systems and various mouse models, these investigators concluded that Wnt pathway activation represses hepatic gluconeogenesis [9,16,22,26,47,48]. We have suggested previously that β-cat/TCF mediates the function of postprandial insulin elevation on repressing hepatic gluconeogenesis [9]. Severe discrepancy in the literature exists regarding the assessment of pancreatic islet *Tcf7l2* knockout mouse models as well [15,21,23,25,49,50].

We suggest that the discrepancy is mainly due to the redundant and overlapping functions of TCF family members and the existence of multiple isoforms for each of the members [1,26]. More importantly, a given TCF is only a “half” effector of Wnt signaling cascade activation. The genetic regulatory function of TCF7L2 or other TCF family members is actually bidirectional, depending on the availability and post-translational modifications of its partner, the β-cat molecule [1]. Thus, to simply knock out a given component of this complicated signaling cascade in mice may not always reveal the exact function of this signaling cascade at physiological and pathophysiological settings.

β-cat serves as the common partner of the 4 TCF family members via interacting with their common β-cat interaction domain. It is well known that β-cat N-terminal phosphorylation (at Ser/Thr 33, 37, 41, 45 residues) lead to its proteasome-mediated degradation, involving glycogen synthase kinase-3 (GSK-3) and another protein kinase casein kinase Iα (CKIα) [51]. The latter one serves as the priming kinase [51]. β-cat can also undergo post-translational modifications on other amino acid residues by various means, and these modifications can be controlled by metabolic hormones, growth factors, and other regulatory molecules, resulting in various functional outcomes [1,8,52]. The utilization of TCF7L2DN, lacking of the β-cat interaction domain, should result in functional knockdown of β-cat/TCF activity in general, avoiding the drawbacks generated by simply knocking out a given TCF family member [1,24,27]. By assessing our *LTCFDN* mouse model, we have reported previously the role of hepatic β-cat/TCF in down-regulating glucose production [26] and present here that hepatic β-cat/TCF also controls hepatic lipid metabolism. More importantly, our current study with the *LTCFDN* mouse model suggests the existence of crosstalk between β-cat/TCF and female hormones.

We show here that without OVX, female mice gained the body weight after HFD feeding for 2 to 4 weeks, followed by the development of resistance to HFD challenge on body weight gain (Fig 5B). This HFD-challenge–induced protection in female mice is clearly attributed to the ovarian gland. When both OVX surgery and HFD challenge were applied, E2 reconstitution effectively restored the metabolic defects in wild-type mice but not in the *LTCFDN* mice (Fig 6). Thus, intact hepatic β-cat/TCF activity is required for systematic E2 administration to protect female subjects in the development of metabolic defects in response to challenge, such as HFD feeding. Our observations thus open a new window in understanding gender differences in the risk of diabetes and other metabolic diseases.

Susceptibilities to metabolic diseases, including T2D, fatty liver disease, and cardiovascular diseases, vary between the genders [53]. With increased life expectancy, women are now spending over 3 decades of life of post menopause, associated with increased susceptibility to metabolic diseases [54]. Menopause hormone therapy has been suggested to delay the onset of T2D [55–57]. Mechanistic explorations to date have mainly implicated the role of estrogens and their receptors (ERα and ERβ) in regulating fuel homeostasis in response to HFD challenge to the suppression of energy intake, adipose tissue accumulation, and improvement of insulin secretion or its sensitivity in the muscle [34–36]. Much less effort has been made on the contribution of the liver, although the nuclear receptor of E2, ERα, was shown to regulate expression of hepatic genes that are involved in de novo lipogenesis (DNL) [58].

In the current study, we observed elevated hepatic and plasma lipid contents in male *LTCFDN* mice, indicating the hepatic function of the Wnt signaling cascade or its effector β-cat/TCF on lipid homeostasis, in agreement with intensive studies on the implication of genetic mutations on the Wnt ligand coreceptor LRP6 in the susceptibility of dyslipidemia [30,59].

Increased hepatic TG content was observed in *LTCFDN* mice at the age of 2 weeks, without the development of defects on other assessed parameters. In this model, *TCF7L2DN* expression is driven by the albumin promoter, and the transgene cannot be detected in the newborn mice [26]. Thus, increased hepatic TG content in these infant mice is directly caused by β-cat/TCF functional knockdown with hepatic TCF7L2DN expression within the first 2 weeks after their birth. This finding is in agreement with previous investigations on familial combined hyperlipidemia with LRP6/Lrp6 mutations [31]. Importantly, our observations revealed for the first time the existence of the crosstalk between the β-cat/TCF signaling cascade and female hormones on hepatic lipid homeostasis, although their convergence has been suggested in cancer and other research fields [32,33].

The function of the β-cat molecule as the Wnt signaling pathway effector was initially recognized in colon cancer studies [42]. Although men and women show comparable frequency in colon cancer development, the incidence rate appears significantly lower in women with menopause hormone therapy [60]. Kouzmenko and colleagues found that ERα and β-cat precipitate within the same immune complexes, and they reciprocally recruited to the cognate response elements in their target promoters and affected their transcriptions [32]. Gupta and colleagues found that in breast cancer cells, β-cat knockdown resulted in reduced ERα levels, associated with reduced ERα target expression [61]. More recently, Wu and colleagues found that estrogen enhances the activity of Wnt signaling cascade during osteogenesis [33]. We revealed here that estrogen facilitates both β-cat Ser675 phosphorylation and TCF expression in hepatocytes and attribute these as the underlying mechanism for repressing hepatic lipogenesis by E2 in mice upon HFD challenge, as E2 also blocked the stimulation of TCF7L2DN on expression of *Mlxipl* and *Srebfl*, as well as their downstream target genes in vitro. More importantly, certain metabolic defects in HFD-fed *LTCFDN* mice with OVX cannot be effectively restored with E2 reconstitution. Our observations obtained with the utilization of the GPER antagonist G15 and the GPER agonist G1 suggest that the effects on β-cat Ser675 phosphorylation and Tcf7l2 expression are at least partially mediated by GPER (Fig 7A–7E). In the rat liver, it has been shown that the GPER/AC/PKA signaling cascade mediates the function of estrogen on cholestasis [38]. Observations made during the past decade caused us to believe that GPER mediates the nongenomic effects of E2 in the liver and elsewhere via increasing cAMP levels [62], consistent with our observation that E2 rapidly activated CREB Ser133 phosphorylation in MPH, followed by increased β-cat Ser675 phosphorylation levels within the 120-minute experimental period. It is worth mentioning that E2 utilized in a previous study on stimulating cAMP elevation a rodent hepatic cell line was as high as 50 μM [38]. We reproduced the results in both HepG2 and MPH with 100 nM E2 (Fig 7B and 7C). At this dosage, E2 treatment can also stimulate *Tcf* gene expression (Fig 4C–4E).

β-cat C-terminal Ser675 phosphorylation was initially reported by Hino and colleagues via PKA activation [63]. Such phosphorylation was then demonstrated in different cell lineages by insulin, GLP-1, and IGF-1. We and others have shown that p21-activated protein kinase (Pak1) mediates the effect of insulin or IGF-1 on β-cat S675 phosphorylation [7,10,11,64,65], while PKA is the downstream protein kinase of the incretin hormone GLP-1 [7,66]. Our current study has expanded the spectrum of hormones that exert their functions via post-translational modifications of β-cat, to include the steroid hormone E2.

It is worth mentioning that the stimulatory effect of E2 treatment on the β-cat/TCF responsive Top-flash activity was relatively modest with the utilization of the 293 naïve cell system (Fig 4G). This, along with the lack of a strong activation on *Axin2* in hepatocytes received 6 hour E2 treatment (Fig 4F) prompted us to suggest that the modest stimulation on β-cat Ser675 itself by E2 is insufficient to activate β-cat/TCF activity robustly. The robust activation can be achieved via the increase on both β-cat Ser675 phosphorylation and TCF expression, occurring in hepatocytes 24 hours after E2 treatment but not in the 293 naïve cell system for conducting our LUC reporter assay (Fig 4G).

Observations obtained in assessing our *LTCFDN* mouse model also prompted us to speculate whether β-cat/TCF is a “missing link” for understanding the paradox of hepatic insulin function and insulin resistance [67–69]. Postprandial insulin elevation represses hepatic gluconeogenesis but increases hepatic lipogenesis. Paradoxically, subjects with insulin resistance show elevations on both gluconeogenesis and lipogenesis. On a LFD without developing insulin resistance, our *LTCFDN* mice showed increased gluconeogenesis as well as lipogenesis, suggesting that β-cat/TCF may serve as a “paradoxical effector” of hepatic insulin signaling.

Fig 7F summarizes our current understanding on the role of E2 on anti-obesity and lipid metabolism in response to Wnt pathway attenuation, which can occur along with aging [1,70,71]. It has been suggested that E2 works with its receptors ERα and ERβ in regulating energy homeostasis in response to HFD challenge via suppressing energy intake and adipose tissue accumulation, increasing insulin secretion, and improving insulin sensitivity, involving the brain, adipose tissue, skeletal muscle, pancreatic islets, and possibly the immune system and other organs [34–36,72,73]. E2 may also utilize the nuclear receptors ERα and ERβ as well as GPER to repress abnormal hepatic lipogenic program activation in response to HFD consumption. This is achieved via facilitating the function of the Wnt signaling pathway effector β-cat/TCF. Thus, our current study expanded the scope of hormonal factors that utilize β-cat/TCF to exert their metabolic functions during adulthood, to include the steroid hormone E2, in addition to previously recognized peptide hormones and growth factors [8] (Fig 7F). Whether E2-stimulated expression of TCF7L2 involves ERα/β as well, or only involves the nongenomic effects of E2 with the participation of GPER in the liver [62], requires further investigations.

Gender differences in the risk, pathophysiology, and complications of T2D and other metabolic diseases have received intensive attention [34]. We learned the impact of testosterone deficiency on the development of visceral obesity and insulin resistance in men, and a recent study revealed a potential mechanism, involving extranuclear actions of the androgen receptor in regulating insulin secretion [74,75]. Our current study suggests that in addition to the regulation of glucose homeostasis via repressing hepatic gluconeogenesis, hepatic β-cat/TCF also participates in lipid homeostasis. Further exploration on how this developmental signaling pathway effector mediates the effect of various metabolic and sex hormones during fasting and after feeding, as well as during health and diseases, may lead to the emergence of novel approaches for metabolic disease treatment and prevention.

## Materials and methods

### Ethics statement

Male and female mice in FVB genetic background were utilized in this study. Animals were handled in strict accordance with good animal practice as defined by national and/or local animal welfare bodies, and all animal work was approved by the Institutional Animal Care and Use Committee of the University Health Network (Protocol number 1560.28). Before conducting the OVX surgery, meloxicam was utilized for analgesia, along with isoflurane. After the surgery, the antibiotic baytril was added to the drinking water for preventing infection.

### Reagents

β-E2-3 benzoate (1,3,5-Estratriene-3,17β-diol, defined as E2 in this study), 3-isobutyl-1-methylxanthine (IBMX), and forskolin were purchased from Sigma Aldrich (Oakville, Canada), while ethanol was utilized as the solvent for the in vitro experiments. The GPER antagonist G15 (CAS no. 1161002-05-6) and the GPER agonist G1 (CAS no. 881639-98-1) were the products of Cayman Chemical (Ann Arbor, MI).

### Animals, OVX and E2 reconstitution

The generation of *LTCFDN* was described previously in which the expression of the human long isoform (75 kDa) TCF7L2DN was under the control of the 2.4 kb mouse serum albumin promoter/enhancer element [26]. Male heterozygous *LTCFDN* mouse were always bred with wild-type female mice for the generation of heterozygous male and female mice and the control wild-type littermates [26]. Bilateral OVX or sham surgery was performed under isoflurane anesthesia. E2 reconstitution was performed via subcutaneous E2 injection (2 μg/mice in peanut oil; 100 μL) every 4 days for 6 weeks, based on the literature [76], or with daily subcutaneous injection for 6 days. OVX was performed with a mid-dorsum approach. In the sham procedure, a similar incision was made and the ovary visualized, but no tissue material was removed.

### Cell cultures

The human hepatic cell line HepG2 (ATCC) and MPHs were prepared and cultured as we have previously described [26]. MPHs were isolated with liver perfusion of 10- to 12-week-old male C57BL6/J mice. HepG2 or MPHs were starved overnight with serum-free DMEM prior to a given treatment. The AD-293 cell line (Agilent Technologies) utilized for adenovirus production was cultured per manufacturer instruction. The 293T cell line (ATCC) was utilized for LUC report gene analysis.

### Metabolic tolerance tests

For IPGTTs and intraperitoneal pyruvate tolerance tests (IPPTTs), both male and female mice were starved for 16 hours prior to the intraperitoneal injection of pyruvate (2 g/kg body weight) or glucose (2 g/kg body weight). For IPITTs, male or female mice were starved for 6 hours prior to the injection of insulin (0.75 U/kg body weight). Assays were conducted as we have previously presented [26].

### Adenovirus infection, RNA isolation, RT-PCR, and real-time RT-PCR

The control adenovirus (Ad-GFP) and TCF7L2DN expressing adenovirus (Ad-TCF7L2DN or Ad-TCFDN) were generated using the AdEasy XL adenovirus vector (Agilent Technologies), as we have previously described [26]. PCR primers and their nucleotide sequences are listed in S1 Table.

### LUC reporter gene analysis

The β-cat/TCF responsive Top-flash reporter gene construct and the control Fop-flash one, as well as the methods for 293 cells transfection and LUC reporter analysis, were described in our previous studies [9,26].

### Western blotting

Whole-cell lysates were prepared for HepG2 cells or MPHs, or mouse liver tissue for western blotting, as we have described previously [9]. Antibodies utilized in this study are listed in S2 Table.

### TG, FFA, and cholesterol measurements

To measure TG contents, liver tissue samples (30 mg) were homogenized in 5% NP-40/H_2_O, followed by centrifugation for supernatant collection. The TG content in the liver supernatant or mouse serum (10 μl) was determined by the utilization of a Serum Triglyceride Determination Kit (TR0100, Sigma Aldrich, Canada). FFA contents were quantified in the liver tissue (10 mg) or serum (2 μl) using the Free Fatty Acid Quantification Kit (Sigma-Aldrich, Canada), following the instructions of the manufacturer. Total cholesterol was measured in serum (2 μl) using the Total Cholesterol E Assay (Wako Diagnostics, USA), following the manufacturer’s instructions. For the measurement of hepatic glycogen content, 20 mg of liver tissue was homogenized in 2 mol/l HCl and boiled for 1 hour followed by neutralization with NaOH. Hydrolyzed glycogen was then determined by measuring glucose using the Glucose (GO) Assay Kit (Sigma Aldrich, Canada).

### Cellular cAMP level measurement

Cellular cAMP levels in the human HepG2 cell line and MPHs were determined utilizing the commercial kit (510040) from Cayman Chemical (Ann Arbor, MI), following the manufacturer’s instruction.

### Statistical analyses

Results are expressed as mean ± SEM. For comparison of 2 groups, the Student *t* test was used. Comparisons between multiple groups were determined by one-way or two-way ANOVA followed by Bonferroni post hoc tests. *P* < 0.05 was considered to indicate a statistically significant difference.

## Supporting information

S1 Fig (Related to Fig 1). **Two-week-old *LTCFDN* mice carry no appreciable abnormalities on their body weight, liver weight, random glucose, serum TG, FFA, or cholesterol levels.** (A) Body weight, (B) liver weight, (C) random glucose levels, (D) serum TG levels, (E) serum FFA levels, and (F) serum cholesterol levels. *p < 0.05. *N* = 3–4 for WT mice and *N* = 8 for LTCFDN mice, regardless of the sex. Values represent mean ± SEM. Underlying numerical values can be found in S1 Data.
(TIF)

S2 Fig (Related to Fig 1). **TCF7L2DN expression results in increased glycogen synthesis.** (A) Hepatic glycogen content in 12-week-old WT (*N* = 4) and LTCFDN (*N* = 6) mice. (B) Higher Gys2 mRNA expression in hepatocytes infected with Ad-TCF7L2DN. *N* = 5 for each treatment in panel B. *p < 0.05. Values represent mean ± SEM. Underlying numerical values can be found in S1 Data.
(TIF)

S3 Fig (Related to Fig 1). **Ad-TCF7L2DN infection increases *ChREBP* (*Mlxipl*) and *SREBP-1c* (*Srebf1*) mRNA levels in the HepG2 cell line.** (A) HA-tagged Ad-TCF7L2DN detection in HepG2 cells. (B) *ChREBP (Mlxipl)* and (C) *SREBP-1c (Srebf1)* mRNA levels after Ad-TCF7L2DN infection in HepG2 cells. *N* = 4 for each treatment in panel B and C. **p < 0.01. Values represent mean ± SEM.
(TIF)

S4 Fig (Related to Fig 3). **TCF7L2DN-induced Mlxipl and Srebf1 elevations were attenuated by estradiol treatment.** (A) ChREBP (Mlxipl) and (B) SREBP-1c (Srebf1) mRNA levels after Ad-TCF7L2DN infection in HepG2 cells. *N* = 8–10 for panel A and B. *p < 0.05. Values represent mean ± SEM.
(TIF)

S5 Fig (Related to Fig 3). **HG-induced *ChREBP* mRNA level can be restored by E2 treatment.** (A) *Chrebp (Mlxipl)* mRNA level after HG and E2 treatment for 16 hours in WT MPH. (B) *Chrebp (Mlxipl)* mRNA level after High glucose (HG) and E2 treatment for 16 hours in WT and LTCFDN MPH. *N* = 3 f or each treatment in panels A and B. Level means without a common letter are statistically different. Values represent mean ± SEM.
(TIF)

S6 Fig (Related to Fig 4). **CREB S133 phosphorylation level was increased after E2 treatment.** (A) CREB S133 phosphorylation level treated with 100 nM E2 for indicated time. (B) Densitometric analysis data of panel A. *N* = 3 for each treatment. ***p < 0.001. Values represent mean ± SEM.
(TIF)

S7 Fig (Related to Fig 5). **The attenuation effect of HFD and OVX on insulin stimulated PKB S473 phosphorylation in skeletal muscles.** (A) PKB S473 phosphorylation in the skeletal muscles. (B) Densitometrical analysis of panel A. *p < 0.05. Values represent mean ± SD.
(TIF)

S8 Fig (Related to Fig 6). **Serum and hepatic FFA levels were comparable in WT and *LTCFDN* mice, regardless of E2 administration or not.** (A) Serum FFA levels. (B) Hepatic FFA levels. *N* = 3–5 for each group in panel A and *N* = 3 for each group in panel B. Values represent mean ± SD.
(TIF)

S9 Fig (Related to Fig 7). **Western blot shows the effect of E2 and G15 treatment on CREB S133 phosphorylation.** Representative blot of 3 independent experiments, with densitometrical analysis results presented in the bottom panel. Cells were pretreated with or without G15 (10 nM) for 45 minutes, followed by E2 (100 nM) or vehicle treatment (as control) for another 120 minutes. *N* = 3 for each treatment. Values represent mean ± STD.
(TIF)

S1 Table (Related to Figs 1, 3, 4, 6 and 7). Primers utilized in this study.
(XLS)

S2 Table (Related to Figs 1, 4, 5, 6 and 7). Antibodies utilized in this study.
(XLS)

S1 Data Numerical values underlying the summary data displayed in this study. (XLS)

## Abbreviations

β-cat: β-catenin
AC: adenylyl cyclase
ACC: acetyl-coA carboxylase
ACSL5: long-chain fatty acid CoA ligase 5
Ad-GFP: GFP-expressing adenovirus
Ad-TCF7L2DN: adenovirus that expresses TCF7L2DN
AUC: area under the curve
Axin2: axis inhibition protein 2
ChREBP: carbohydrate-responsive element-binding protein
CKIα: casein kinase Iα
CREB: cAMP response element-binding protein
DN: dominant negative
DNL: de novo lipogenesis
E2: estradiol
EHHADH: enoyl-coA hydratase and 3-hydroxyacyl coA dehydrogenase (also known as peroxisomal L-bifunctional enzyme)
ELISA: enzyme-linked immunosorbent assay
ERα/β: estrogen receptor α/β
Fas: fatty acid synthase
FFA: free fatty acid
GAPDH: glyceraldehyde-3-phosphate dehydrogenase
GFP: green fluorescence protein
GIP: gastric inhibitory polypeptide
GLP-1: glucagon-like peptide-1
GPER: G-protein-coupled estrogen receptor
GSK-3: glycogen synthase kinase-3
GWAS: genome-wide association study
Gys2: glycogen synthase 2
HA: hemagglutinin (tag)
HE: hematoxylin–eosin
HFD: high-fat diet
IBMX: 3-isobutyl-1-methylxanthine
IGF-1: insulin-like growth factor-1
IGF-1R: insulin-like growth factor 1 receptor
IPGTT: intraperitoneal glucose tolerance test
IPITT: intraperitoneal insulin tolerance test
IPPTT: intraperitoneal pyruvate tolerance test
Lef1: lymphoid enhancer binding factor 1
LFD: low-fat diet
LRP6: lipoprotein receptor-related protein 6
LUC: luciferase
ME1: cytosolic malic enzyme 1
MPH: mouse primary hepatocyte
OVX: ovariectomy
p-ACC: phosphorylated acetyl-coA carboxylase
Pak1: p21-activated protein kinase 1
PKA: protein kinase A
PKB: protein kinase B
qRT-PCR: quantitative reverse transcription polymerase chain reaction
SCD-1: stearoyl-coA desaturase-1
SNP: single nucleotide polymorphism
SREBP-1c: sterol regulatory element-binding protein 1-c
T2D: type 2 diabetes
TCF: T cell factor
TCF7L2: transcription factor 7 like 2 (Tcf7l2 in rodents)
TCF7L2DN: dominant-negative TCF7L2
TG: triglyceride
WT: wild type
