# Supplementary figures and images for: The developmental Wnt signaling pathway effector β-catenin/TCF mediates hepatic functions of the sex hormone estradiol in regulating lipid metabolism
Source: PLoS Biol. 2019 Oct 7;17(10):e3000444. doi: 10.1371/journal.pbio.3000444 (PMC6797220; doi:10.1371/journal.pbio.3000444)

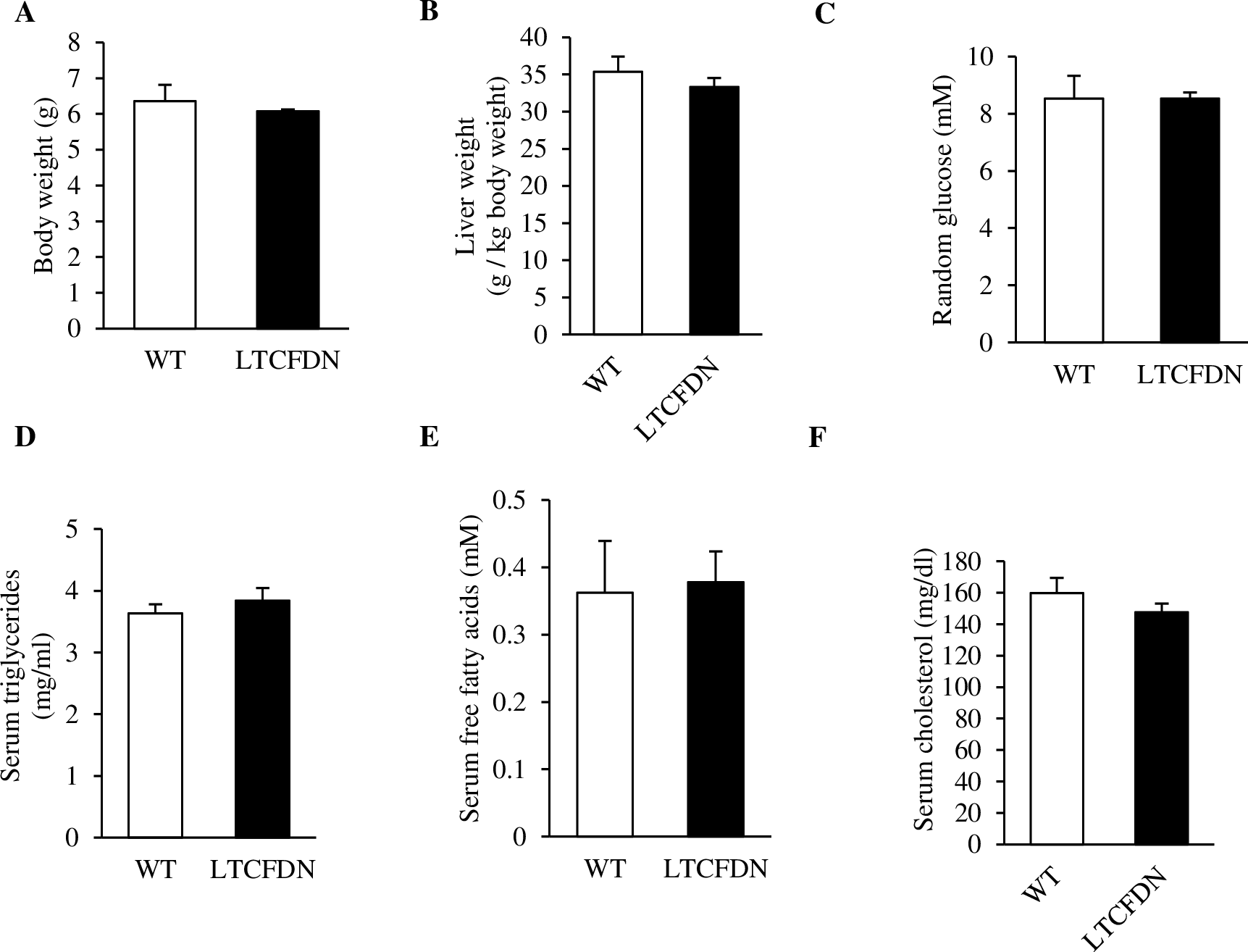

Supplement: S1 Fig — Two-week-old LTCFDN mice carry no appreciable abnormalities on their body weight, liver weight, random glucose, serum TG, FFA, or cholesterol levels. (A) Body weight, (B) liver weight, (C) random glucose levels, (D) serum TG levels, (E) serum FFA levels, and (F) serum cholesterol levels. *p < 0.05. N = 3–4 for WT mice and N = 8 for LTCFDN mice, regardless of the sex. Values represent mean ± SEM. Underlying numerical values can be found in S1 Data. (TIF) [file pbio.3000444.s001.tif]

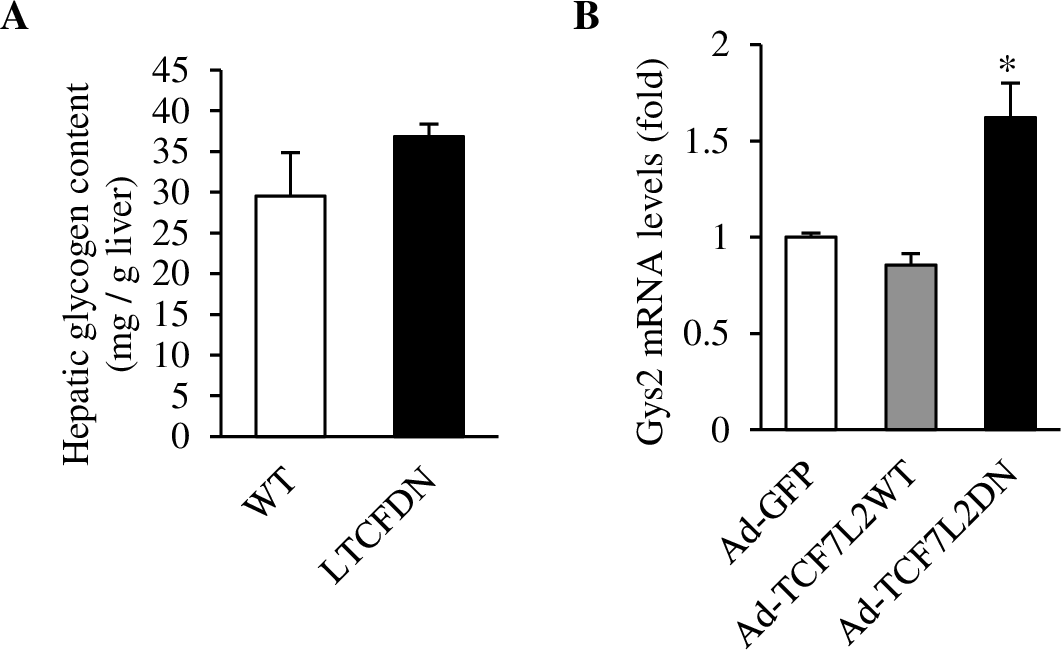

Supplement: S2 Fig — TCF7L2DN expression results in increased glycogen synthesis. (A) Hepatic glycogen content in 12-week-old WT (N = 4) and LTCFDN (N = 6) mice. (B) Higher Gys2 mRNA expression in hepatocytes infected with Ad-TCF7L2DN. N = 5 for each treatment in panel B. *p < 0.05. Values represent mean ± SEM. Underlying numerical values can be found in S1 Data. (TIF) [file pbio.3000444.s002.tif]

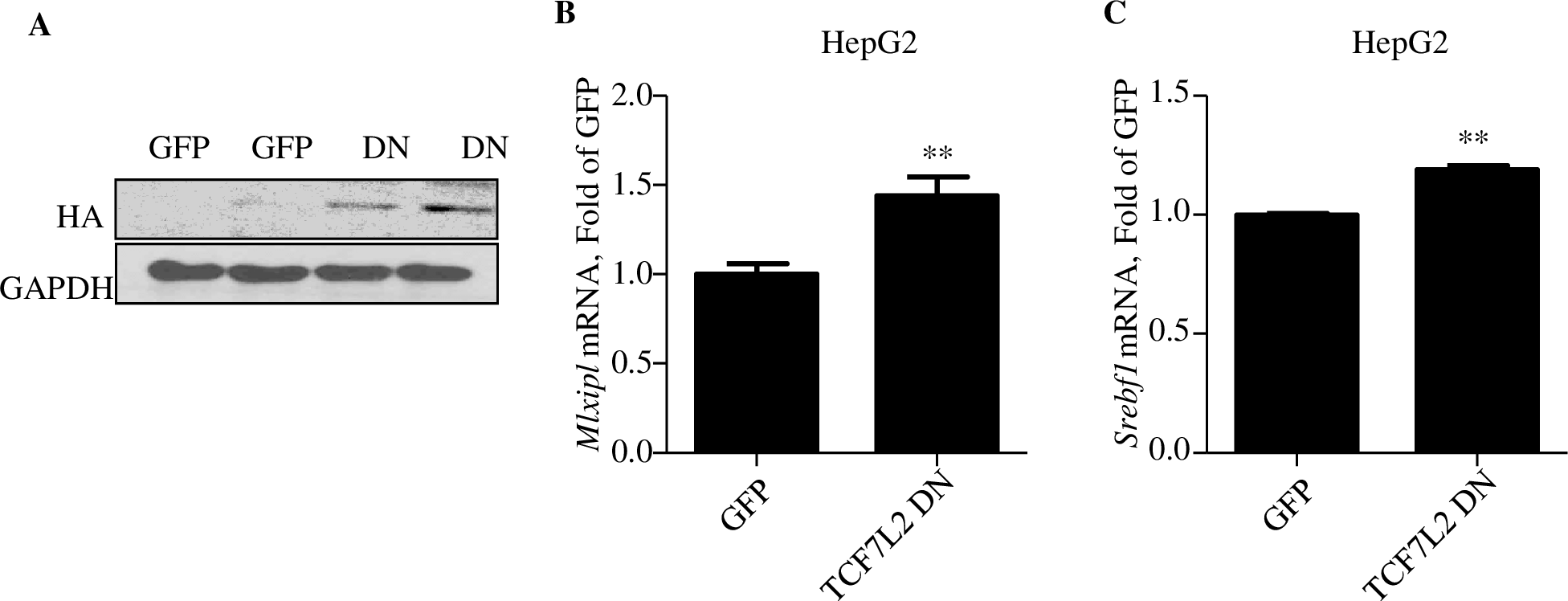

Supplement: S3 Fig — Ad-TCF7L2DN infection increases ChREBP (Mlxipl) and SREBP-1c (Srebf1) mRNA levels in the HepG2 cell line. (A) HA-tagged Ad-TCF7L2DN detection in HepG2 cells. (B) ChREBP (Mlxipl) and (C) SREBP-1c (Srebf1) mRNA levels after Ad-TCF7L2DN infection in HepG2 cells. N = 4 for each treatment in panel B and C. **p < 0.01. Values represent mean ± SEM. (TIF) [file pbio.3000444.s003.tif]

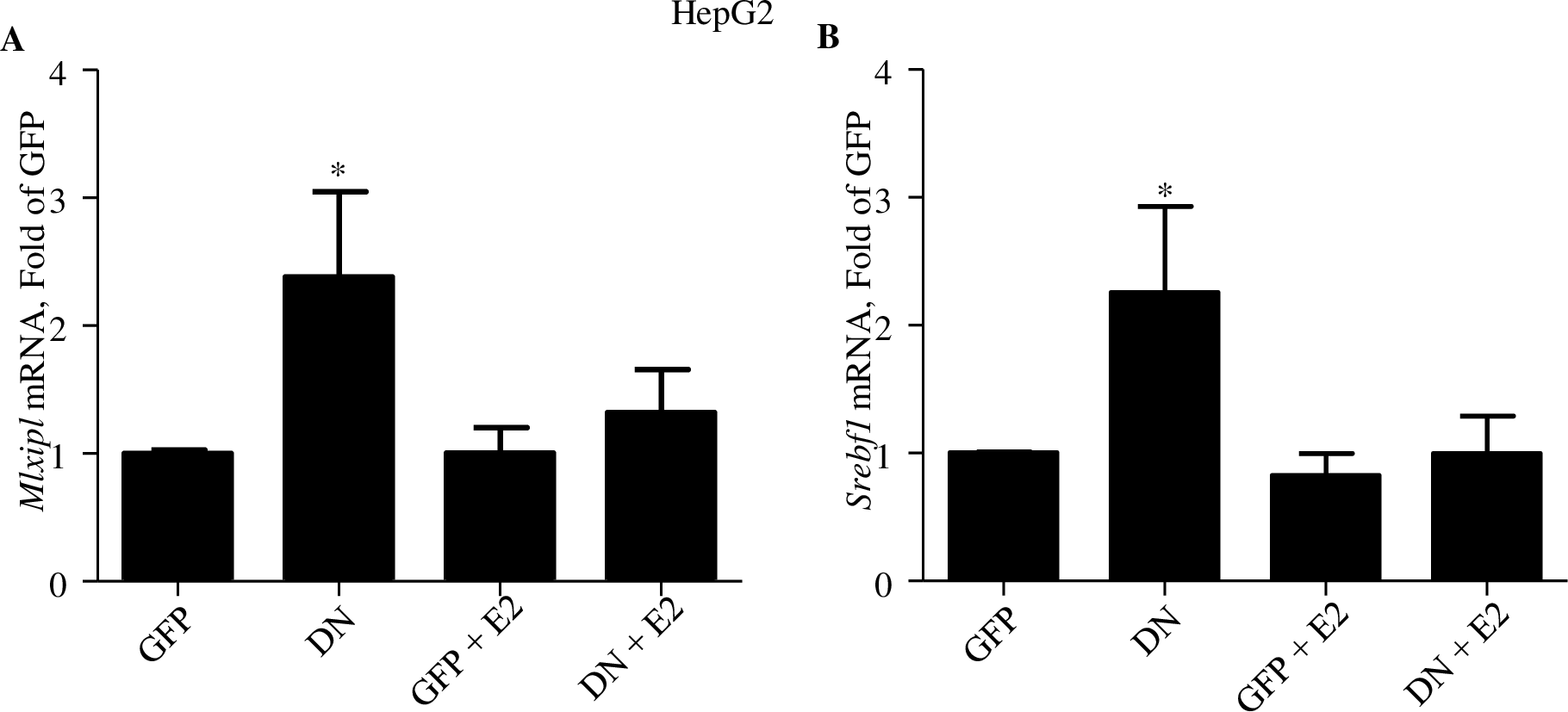

Supplement: S4 Fig — TCF7L2DN-induced Mlxipl and Srebf1 elevations were attenuated by estradiol treatment. (A) ChREBP (Mlxipl) and (B) SREBP-1c (Srebf1) mRNA levels after Ad-TCF7L2DN infection in HepG2 cells. N = 8–10 for panel A and B. *p < 0.05. Values represent mean ± SEM. (TIF) [file pbio.3000444.s004.tif]

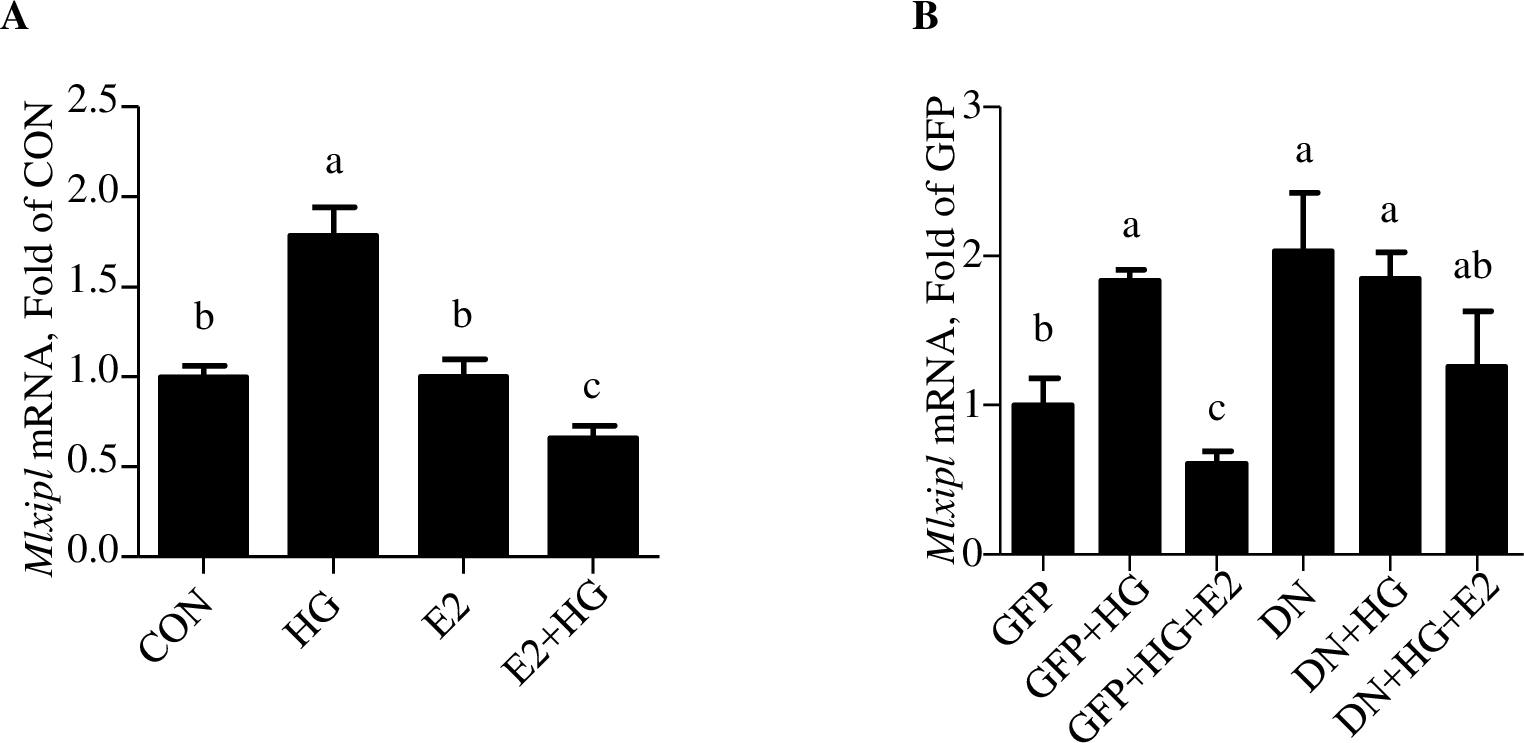

Supplement: S5 Fig — HG-induced ChREBP mRNA level can be restored by E2 treatment. (A) Chrebp (Mlxipl) mRNA level after HG and E2 treatment for 16 hours in WT MPH. (B) Chrebp (Mlxipl) mRNA level after High glucose (HG) and E2 treatment for 16 hours in WT and LTCFDN MPH. N = 3 f or each treatment in panels A and B. Level means without a common letter are statistically different. Values represent mean ± SEM. (TIF) [file pbio.3000444.s005.tif]

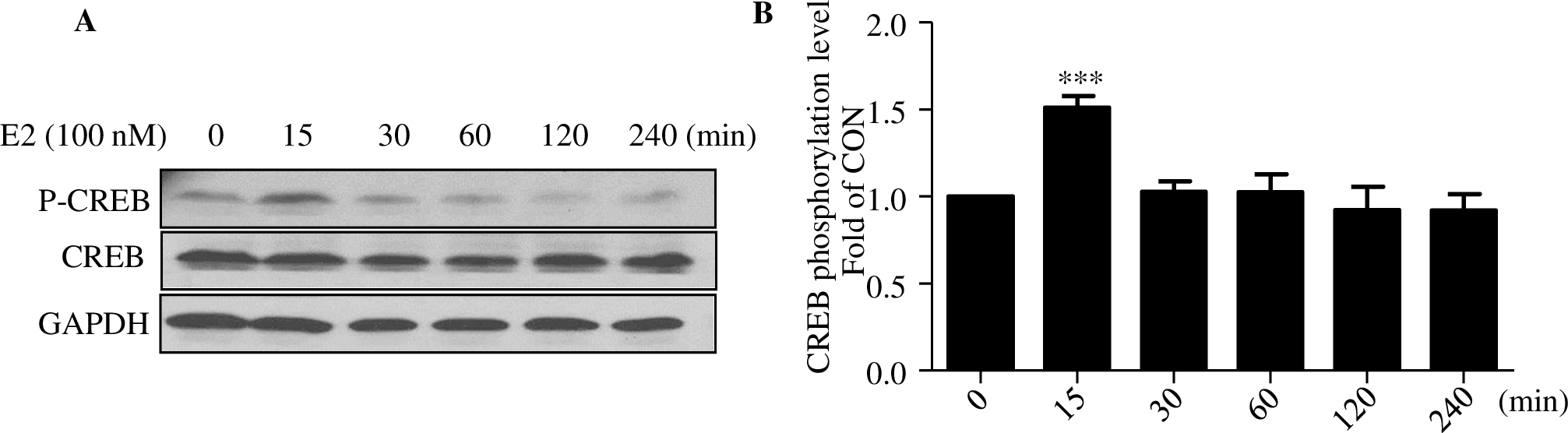

Supplement: S6 Fig — CREB S133 phosphorylation level was increased after E2 treatment. (A) CREB S133 phosphorylation level treated with 100 nM E2 for indicated time. (B) Densitometric analysis data of panel A. N = 3 for each treatment. ***p < 0.001. Values represent mean ± SEM. (TIF) [file pbio.3000444.s006.tif]

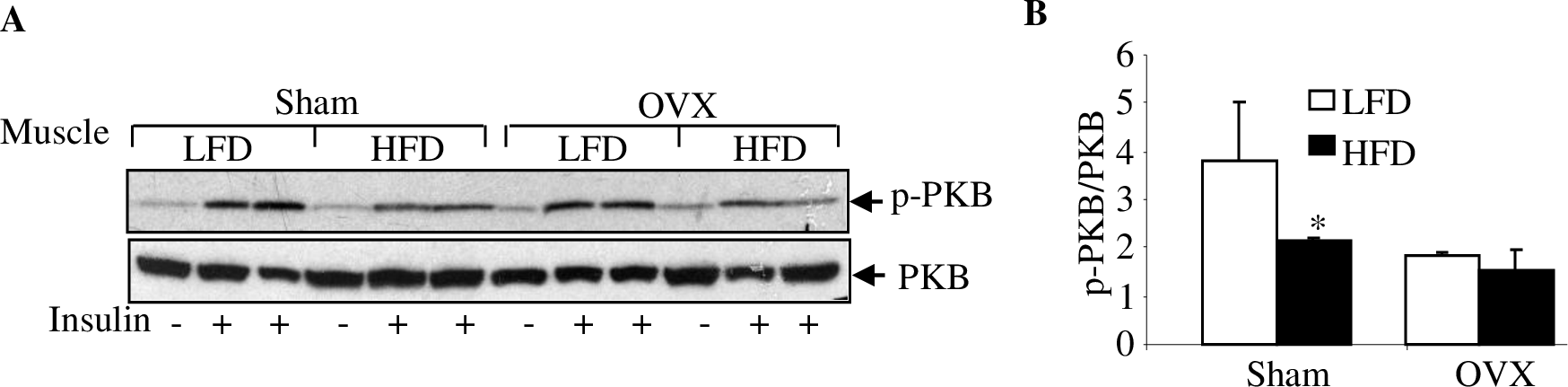

Supplement: S7 Fig — The attenuation effect of HFD and OVX on insulin stimulated PKB S473 phosphorylation in skeletal muscles. (A) PKB S473 phosphorylation in the skeletal muscles. (B) Densitometrical analysis of panel A. *p < 0.05. Values represent mean ± SD. (TIF) [file pbio.3000444.s007.tif]

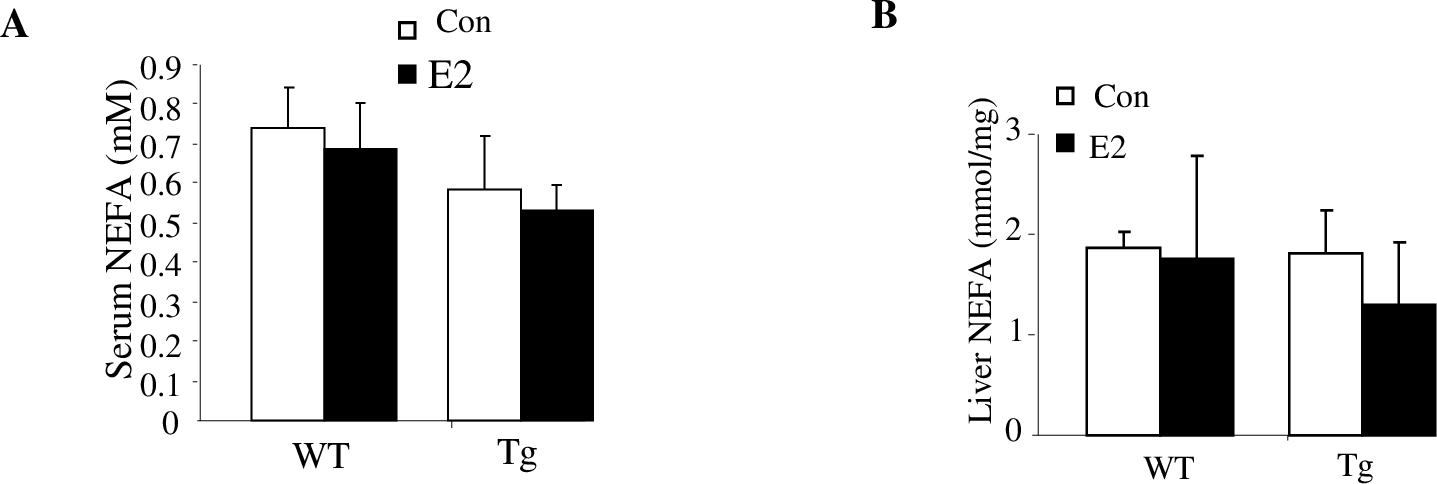

Supplement: S8 Fig — Serum and hepatic FFA levels were comparable in WT and LTCFDN mice, regardless of E2 administration or not. (A) Serum FFA levels. (B) Hepatic FFA levels. N = 3–5 for each group in panel A and N = 3 for each group in panel B. Values represent mean ± SD. (TIF) [file pbio.3000444.s008.tif]

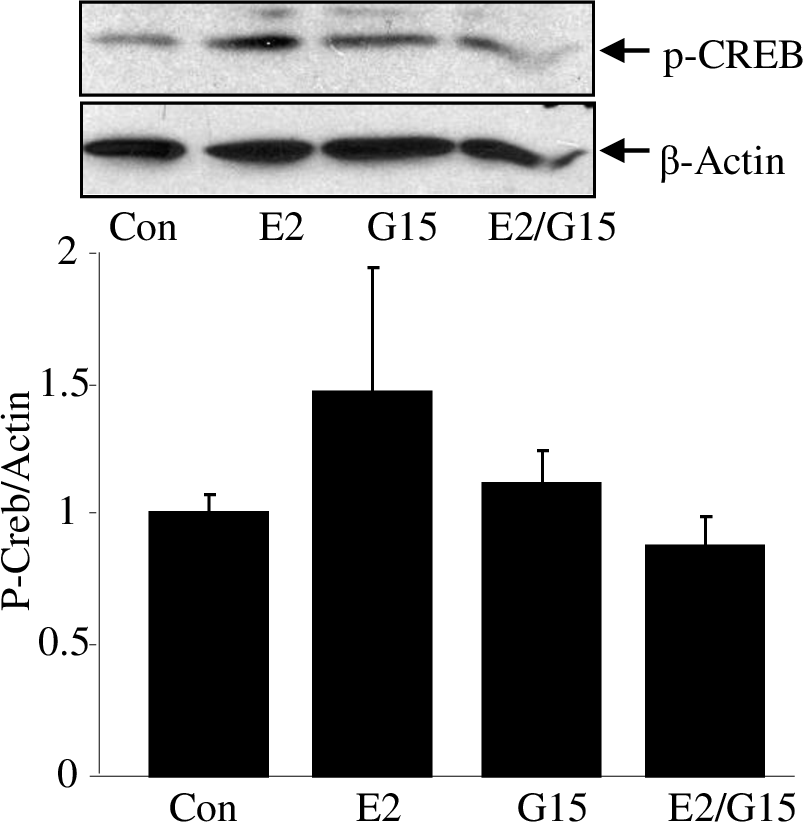

Supplement: S9 Fig — Western blot shows the effect of E2 and G15 treatment on CREB S133 phosphorylation. Representative blot of 3 independent experiments, with densitometrical analysis results presented in the bottom panel. Cells were pretreated with or without G15 (10 nM) for 45 minutes, followed by E2 (100 nM) or vehicle treatment (as control) for another 120 minutes. N = 3 for each treatment. Values represent mean ± STD. (TIF) [file pbio.3000444.s009.tif]
